# The Global Polarity of Alcoholic Solvents and Water – Importance of the Collectively Acting Factors Density, Refractive Index and Hydrogen Bonding Forces

**DOI:** 10.1002/open.202200140

**Published:** 2022-10-25

**Authors:** Stefan Spange, Nadine Weiß, Thomas G. Mayerhöfer

**Affiliations:** ^1^ Institute of Chemistry Chemnitz University of Technology Straße der Nationen 62 09111 Chemnitz Germany; ^2^ Leibniz Institute of Photonic Technology Albert-Einstein-Straße 9 07745 Jena Germany; ^3^ Institute of Physical Chemistry and Abbe Center of Photonics Friedrich Schiller University Helmholtzweg 4 Jena 07743 Germany

**Keywords:** alcohol, density, polyol, refractive index, solvatochromism

## Abstract

The *D*_HBD_ quantity represents the hydroxyl group density of alcoholic solvents or water. *D*_HBD_ is purely physically defined by the product of molar concentration of the solvent (*N*) and the factor Σn=n×*f* which reflects the number n and position (*f*‐factor) of the alcoholic OH groups per molecule. Whether the hydroxyl group is either primary, secondary or tertiary is taken into account by *f*. Σn is clearly linearly correlated with the physical density or the refractive index of the alcohol derivative. Relationships of solvent‐dependent UV/Vis absorption energies as *E*_T_(30) values, ^129^Xe NMR shifts and kinetic data of 2‐chloro‐2‐methylpropane solvolysis with *D*_HBD_ are demonstrated. It can be shown that the *E*_T_(30) solvent parameter reflects the global polarity of the hydrogen bond network rather than specific H‐bond acidity. Significant correlations of the log *k*_1_ rate constants of the solvolysis reaction of 2‐chloro‐2‐methylpropane with *D*_HBD_ show the physical reasoning of the approach.

## Graphical abstract

**The density of the hydroxyl groups** and the molar concentration primarily determine the global polarity of the alcohols and water. To identify this effect, the polar dissolved Reichardt dye (*E*_T_(30) value), the nonpolar xenon atom (^129^ Xe chemical shift) and the solvolysis of the weakly dipolar 2‐chloro‐2‐methylpropane (lg*k*_1_ of solvolysis) are introduced, discussed and compared as suitable probes.

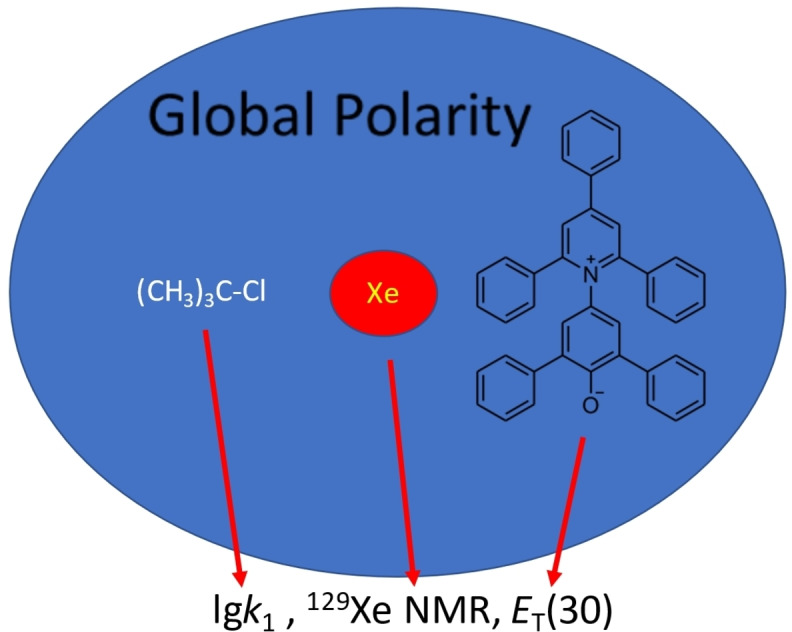

## Introduction

The complexity of the situation in interpreting solvent properties is particularly pronounced in the case of alcoholic solvents and water.[^1^, ^2^, ^3^, ^4^] The physical properties and liquid structures of alcohols are many‐faceted. These are naturally determined by their molecular weight, the numbers and positions of OH groups on the C‐skeleton and the degree of branching of the alkyl chain.^[1]^ Therefore, alcohol derivatives can be classified in many ways. For example, methanol, ethanol, 1‐propanol, 1‐butanol and n‐alkanol belong to a homologous series based on the number of carbon atoms in the primary alcohols. An alcohol family refers to alcohol derivatives with the same number of carbon atoms; for example, 1‐propanol, 2‐propanol, propanediols and glycerol belong to the C_3_ family as discussed in this article. The classification into primary, secondary and tertiary alcohol classes or mono‐ and polyhydric alcohols is also well documented. Thus, both very similar and significantly different properties are observed for monohydric and polyhydric alcohols. In the course of this study, it will become clear that various distinctions have to be taken into account and play a special role.

**Figure 1:**
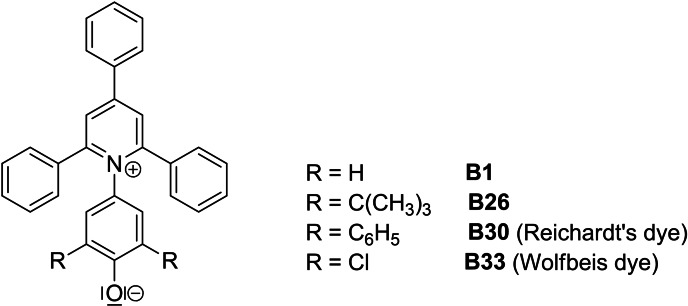
Solvatochromic dyes of the Reichardt type: 4‐(2,4,6‐triphenylpyridinium‐1‐yl)phenolate (**B1**), 2,6‐di‐*tert‐*butyl‐4‐(2,4,6‐triphenylpyridinium‐1‐yl)phenolate (**B26**), 2,6‐diphenyl‐4‐(2,4,6‐triphenylpyridinium‐1‐yl)phenolate (**B30**)^[5e]^ and 2,6‐dichloro‐4‐(2,4,6‐triphenylpyridinium‐1‐yl)phenolate (**B33**).^[6]^

Alcohols and water are normally characterized as HBD (hydrogen‐bond‐donating) solvents, because the O−H bond is able to donate a positively polarized hydrogen atom towards a solute.[^1^, ^2^, ^3^, ^4^] It has, however, been recognized that the overall polarity (global polarity) of the alcohols in the volume of the solvent exerts a much greater influence than the hydrogen bond strength of the monomeric OH group to a particular solute.^[2]^ In particular, the empirical *E*_T_(30) parameter for the polarity of the solvent is very sensitively influenced by the structure of the alcoholic solvents.^[5]^ The original *E*_T_(30) parameter is defined as the molar absorption energy of 2,6‐diphenyl‐4‐(2,4,6‐triphenyl‐1‐pyridinium)phenolate (**B30**, see (Figure 1) expressed in kcal mol^−1^, measured in a given solvent [Eq. 1].^[5b]^


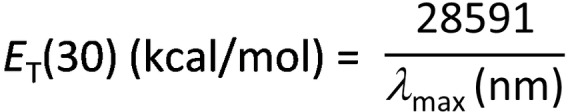




In this context, the term “polarity” has an uncertain meaning.^[5a]^ There are numerous studies on the origin of the UV/Vis band shift of **B30** and related dyes (Figure 1) as a function of solvent properties.[^5c^, ^6^]

In this paper, we will consider some alternative interpretations regarding the non‐chemical effect of alcohols and related HBD solvents on the *E*_T_(30) value. In fact, the interpretation of UV/Vis band shifts and intensity changes of dyes as a function of solvent nature is a phenomenon that has been studied for more than 150 years and these interpretations underwent several changes. First, Kundt formulated a rule in 1871 according to which the UV/Vis absorption of the dye is shifted towards the red the stronger the dispersion of the solvent in this spectral range is.^[6]^

The almost unanimous opinion at the time was that Kundt's rule cannot be applied if “chemical influences are obvious”. The physical basis of Kundt's rule was not understood at that time. Recently, it could be demonstrated that electromagnetic coupling of electronic excitations of solutes **B1**, **B26**, and **B30**, respectively, (Figure 1) with those of the solvent plays a major role.^[8a]^ In the simplest case, this coupling is described by the frequency dependent Lorentz‐Lorenz relation, which predicts red shifts and also intensity changes with the same order of magnitudes like those that are experimentally observed.[^8b^, ^8c^, ^8d^]

In addition, new important physical research results on the UV/Vis spectroscopic properties of a special solvatochromic pyridinium‐1‐yl‐phenolate dye have recently reported, which are essential for the correct interpretation of the *E*_T_(30) values.^[9]^ This current finding that HBD solvent molecules with pyridinium‐1‐yl‐phenolate dyes form the actual solvatochromic complex is taken into account in this work to reinterpret *E*_T_(30).

Section A of the Supporting Information summarises some aspects on “global polarity” from the literature showing the problem when different concepts [*(*Lorentz‐Lorenz, Debye equation, linear solvation energy relations (LSER)] are considered alternatively.[^10^, ^11^, ^12^, ^13^, ^14^, ^15^, ^16^, ^17^, ^18^, ^19^, ^20^]

According to the Lorentz‐Lorenz or Debye equation, the influence of the “global polarity” of the solvent volume will be analyzed in terms of their molar concentration *N*.[^11^, ^13^, ^14^, ^20^] *N* is an important physical quantity that represents the relationship between density *ρ* and molar mass *M*, regardless of its state of aggregation [Eq. (2)]. The effect of *N* on the polarity of alcoholic solvents was first discussed by Langhals.^[11a]^ Unfortunately, these crucial aspects have been underestimated in the literature so far.[^5^, ^19^] Recently, it has been shown that the solvent‐dependent |C≡N stretching vibration of indole‐isonitrile derivatives measured in different alcohols also correlates with the *N* of the alcohols in a complex way.^[21]^ Furthermore, the HBD group density (*D*_HBD_) of the (alcoholic) solvent volume also depends on the number of OH groups per solvent molecule^[21b]^ *D*_HBD_ is thus defined as the product of *N* and the number (n) of HBD groups in the single solvent molecule [Eq. 2].^[21b]^

(2) $$D{}_{\mathrm{HBD}}=nN=n\rho /M=n/V{}_{m}$$

Where *V*_m_ is the molar volume. With alcohols, the situation is straightforward because the OH groups are the only HBD unit. Thus, *D*_HBD_ corresponds to the density of OH groups. Simply put, n in Equation (2) was set to 2 for 1,2‐ethanediol and 3 for glycerol by the authors of Ref. [21b]. The physical justification and limitation of Equation (2) is explained in a more detailed way in the Supporting Information (2.1: Aspects and solvent size and molar volume (*V*_m_) as a function of structure of alcoholic solvents).

The results in Refs. [21,22] clearly support the hypothesis of several studies[^11^, ^13b^, ^20^] that the number of OH groups per volume (hydroxyl group density) determines the influence of the alcoholic solvents on a measurand and not the HBD‐strength (solvent acidity) of the individual functional OH group. This result is in full agreement with the results of our earlier study, according to which the electromagnetic coupling of the oscillators of solvent and solute determine the measured value.^[8a]^ Therefore, in this work, we want to refine the so‐called *D*_HBD_ quantity from Ref. [21b] which allows an extension of our concept in Ref. [13b] as a quantity to describe the global polarity of alcoholic solvents.

The limitation of *D*_HBD_ concerns the positions and arrangement of the OH substituents in a given volume, which determine their actual effectiveness, as shown by several physical studies on different types of alcohols.[^1^, ^22^, ^23^, ^24^, ^25^] The electromagnetic coupling of the solvent's oscillators located in the UV/Vis region with those of the dye plays an important role.^[26]^ This aspect is treated in particular in this work, since the position of the OH group and the carbon skeleton of the alcohol derivative also strongly influence the oscillator strength. This was also clearly shown in the *E*_T_(30) parameter as a function of the static dielectric constant ϵ_r_ for primary compared to secondary and tertiary alcoholic solvents: A different relationship results for each solvent class.^[5e]^

**Scheme 1:**
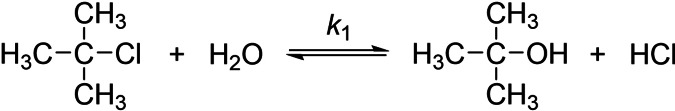
Solvolysis reaction of 2‐chloro‐2‐methylpropane.

The objective of this study is to investigate the correlations of *D*_HBD_ with solvent‐dependent physical properties of three different probe solutes from the literature. Three completely different physical methods are used to investigate the solvent‐dependent measurand. The molar UV/Vis absorption energy of **B30** (Figure 1, Equation (1); note that we keep this term, although we are fully aware of the fact that peak positions in absorbance spectra can, in general, not be directly identified as the energy difference between electronic states as implied by this terminology), the ^129^Xe NMR chemical shift δ,^[27]^ and the first order rate constant *k*_1_ of the solvolysis of 2‐chloro‐2‐methylpropane (tertiary butyl chloride, ^*t*^BuCl (Scheme 1).^[28]^

The probes selected are significantly different in their physical property: the highly dipolar **B30**,^[5]^ the nonpolar Xe atom,^[27]^ and the “transition state” of ^*t*^BuCl in a solvolysis reaction.^[28]^ Despite the strong differences in their static dipole moment μ (in Debye, D) **B30** (μ=15 D)≫^t^BuCl (μ=2.13 D)>Xe (μ=0), each of the three probes has a different, remarkably solvent‐dependent physical property, which is used as a criterion for evaluation. The reason for the strong solvent influence upon a nonpolar solute is the strong coupling of dipolar and polarizability effects.^[29]^ This scenario occurs for polar **B30** as well as for non‐polar solutes as Xe and in the transition of the weakly polar reactant ^*t*^BuCl to the reactive (highly dipolar) intermediate in a polar reaction.

Xe has no static dipole moment. Its (induced) dipole moment depends on the interacting partner^.[29]^ Xe is not able to form directional hydrogen bonds with moderately strong HBD solvents such as alcohols or carboxylic acids. However, there are also some studies showing that weak H bonds are formed in the case of stronger acids with p*K*_a_<0.[^30^, ^31^] Therefore, Xe seems to be the ideal counterpart to **B30** to exclusively test the dispersion interactions with alcohols. The strongly solvent‐dependent chemical shift δ of the ^129^Xe NMR signal in alcohols and water will be evaluated in relation to *D*_HBD_.

The ^*t*^BuCl molecule is a moderately polar solute with a static dielectric constant ϵ_r_=10.95, but it spontaneously reacts with water to form 2‐methyl‐propane‐2‐ol (^*t*^BuOH) and HCl (Scheme 1).^[28]^

The strongly solvent‐dependent *k*_1_ values of the s_n_ 1 solvolysis reaction of various 2‐halogeno‐2‐methylpropanes have become established as suitable criteria for classifying the polarity effects of solvents.[^5d^, ^28^, ^32^, ^33^, ^34^] Winstein's *Y* value was thus the first empirically determined polarity parameter of solvents derived from *k*_1_ of the solvolysis of ^*t*^BuCl [Eq. 3].^[28b]^

(3) $$\mathrm{lg}(k{}_{1}/k{}_{o})=m\;\cdot \;Y$$

With *Y* representing the ionizing power of the solvent referenced with *Y*=0 for 80 % aqueous ethanol (m=1) with *k*_o_ at 25 °C. In particular, water as a solvent (p*K*_a_=15.7) dramatically accelerates this reaction, much more so than more strongly acidic solvents such as formic acid (p*K*_a_=3.75), 1,1,1,3,3,3‐hexafluoroisopropanol (HFIP) (p*K*_a_=9.6) or 2,2,2‐trifluoroethanol (TFE) (p*K*_a_=12.4). According to arguments in the literature, the reaction rate is strongly determined by the solvents’ acidity.[^33^, ^34^] So far, there is no conclusive explanation for this discrepancy between p*K*_a_ and ln*k*_1_ In Ref. [11b], the solvolysis kinetics of ^*t*^BuCl in ethanol‐water mixtures were interpreted in terms of composition in relation to the *E*_T_(30) polarity parameter. However, the interplay of enthalpic (energetic) and entropic effects on the rate constant is difficult to quantify in terms of solvent composition.^[28c]^ The results in Ref. [11b] thus are a motivation to evaluate the correlation of lg*k*_1_ with *N* or *D*_HBD_.

The fluorinated alcohols HFIP and TFE have special characteristics. New findings on the liquid structure of HFIP and TFE from the literature are included in the discussion at appropriate points with references. Moreover, due to critical comments and comparative considerations in recent works,[^35^, ^36^] we also feel compelled to consolidate and considerably extend the analysis of alcoholic solvents of our earlier study.^[13b]^

The aim of this work is not to define a new solvent parameter, but to provide a physically correct understanding of known solvent‐dependent measurement data in relation to the physical properties (molar mass, density, refractive index) of the solvent, which will be discussed comparatively as alternative approaches. In this work, we prefer to work with pure solvents and only selectively consider solvent mixtures in the kinetics of the solvolysis of ^*t*^BuCl.

## Results and Discussion

For reasons of comprehensibility, the scientific arguments for using Equation (2) as the basis are briefly summarized in the Supporting Information (Section 2.1), which documents relationships of the molar volume *V*_m_=1/*N* as a function of solvent molecule size (Figures S1 and S2).[^37^, ^38^, ^39^, ^40^]

The refined *D*_HBD_ concept and an alternative interpretation of the *E*_T_(30) parameter will be demonstrated first as it is of fundamental importance for the understanding of the *D*_HBD_ quantity. The plotting of *E*_T_(30) as a function of *N* was performed for a set of 43 alcoholic HBD solvents including monohydric primary, secondary, tertiary, dihydric and polyhydric alcohol as well as monohydric amino alcohols and substituted derivatives (see Figure S3a). Data used from literature are given in Tables S1 and S2 in the Supporting Information. The *E*_T_(30) value for 1,2,4‐butanetriol was additionally measured for this work.

The *E*_T_(30) parameter for primary monohydric non‐branched alcohols, including water, is an approximately linear function of the molar concentration *N* of the solvent as shown in Equation (4) from Ref. [13b].

(4) $$E{}_{T}\left(30\right)=314.4\;\cdot \;N+46n=13\;(\mathrm{water}\;\mathrm{and}\;C{}_{1}\;\mathrm{to}\;C{}_{12}\;n\text{-}\mathrm{alcohols}),\;r=0.994$$

If water is excluded, as done in this work, we obtain Equation (5), which has a particularly high correlation quality. This is largely consistent with results of earlier studies by Langhals and Bentley.[^11a^, ^20^] The exclusion of water is motivated by the study of Świergiel which showed both the relationship and the difference of the water properties to the series of primary alcohols.^[23]^ In contrast to Ref. [11a], methanol does fit well in this linear relation.

(5) $$E{}_{T}\left(30\right)=383.19\;\cdot \;N+45.66n=12\;(C{}_{1}-C{}_{12}\;n\text{-}\mathrm{alkanols}),\;r=0.998$$

This result is a clear indication that the *E*_T_(30) parameter for primary alcohols is essentially determined by volume properties of the solvent and not by specific hydrogen bonds, which is consistent with the theory of electrostatic coupling between solvent and solute.[^8a^, ^41^]

The overall correlation of *E*_T_(30) as function of *N* for all considered HBD solvents (n=43; Equation (S8), Supporting Information) is not very good (*r*=0.608), but individual separate correlations are recognizable.

Basically, it can be deduced that all alcohols with higher *E*_T_(30) values than expected according to Equation (5) have a higher HBD group density than indicated by *N*. For the alcohols below the straight line of Equation (5), the situation is reversed. They have a lower HBD group density in relation to *N*. The alcohol derivatives which do not fit in the primary alcohol correlation can be distinguished as follows:

- Secondary 2‐alkanols are arranged almost parallel to the primary ones, but show lower *E*_T_(30) values [see Equation (6)].
- Tertiary alcohols [such as ^*t*^BuOH and 3‐ethyl‐2,4‐dimethyl‐3‐pentanol (EDMP)] as well as 2,4‐dimethyl‐3‐pentanol (DMP) clearly show lower *E*_T_(30) values, but an own separate relationship (open symbols) as marked in Figure 2
  **Figure 2:**
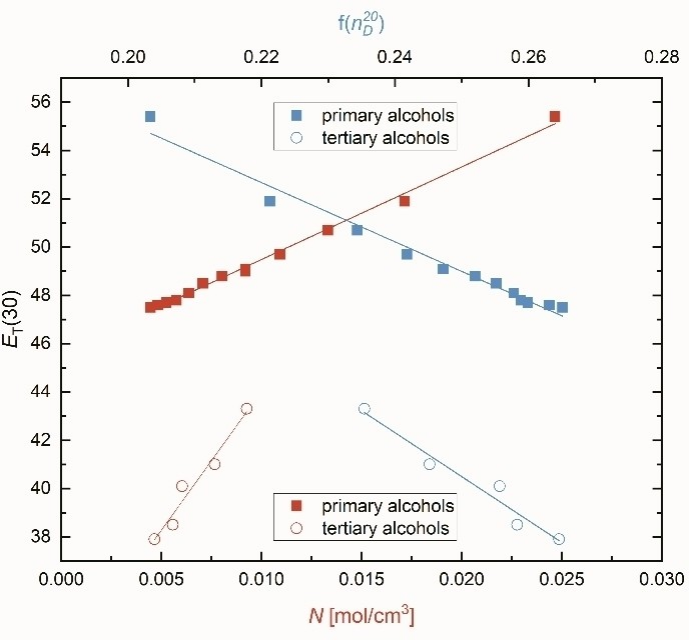
Correlations of E_T_(30) as a function of molar concentration N (red) and refractive index f($n_{D}^{20}$ )=[($n_{D}^{20}$ )^2^−1]/[($n_{D}^{20}$ )^2^+2] (blue) for primary alcohols (filled symbols) and tertiary alcohols (open symbols).
  [see Equation (7)].
- Polyhydric alcohols show larger *E*_T_(30) values than expected from Equation (5).
- TFE and HFIP also show significantly larger *E*_T_(30) values than expected.

to a)6

(6) $$E{}_{T}\left(30\right)=607\;\cdot \;N+40.5n=4\;(\mathrm{secondary}\;\mathrm{alcohols}),\;r=0.954$$

to b)7

(7) $$E{}_{T}\left(30\right)=1151\;\cdot \;N+32.52n=5\;(\mathrm{tertiary}\;\mathrm{and}\;\mathrm{sterically}\;\mathrm{shielded}\;\mathrm{alcohols}),\;r=0.981$$

The different linear correlations are probably due to the different sensitivity of *E*_T_(30) depending on the refractive index $n_{D}^{20}$ of the respective alcohol. $n_{D}^{20}\;$ is the refractive index of the solvent measured at 589 nm [Na−D‐line]). If the electronic absorptions are situated in the far UV region, then $n_{D}^{20}$ is a measure of the overall oscillator strength of the electronic transitions, that is, it is proportional to the number of C atoms. In addition, the position of the OH group also seems to be characteristic.[^1^, ^3^, ^23^, ^24^, ^25^] Thus, the decrease of $n_{D}^{20}$ as a function of *N* is more pronounced for tertiary alcohols than for primary or secondary ones indicating a higher oscillator strength and/or a stronger coupling of the solvent oscillators with those of the solute and consequently a stronger redshift in accordance with the previous study.^[8a]^ The high significance of the correlations is impressive and indicates reliable relationships (Figure 2).

The following consideration is particularly important because it represents a fundamental context. Currently, it has been demonstrated that the molar absorption energy 1/*λ*_max_ [related to *E*_T_(30), Equation (1)] and the molar absorption coefficient of **B1**, **B26** and **B30**, respectively, are related to each other.^[8a]^ Analysing these relationships, the quotient of the molar absorption energy 1/λ_max_ (in cm^−1^) and the molar absorption coefficient ϵ (in cm^2^/mol) [Eq. (8a) or (8b)] correspond to the *N*, because the ratio 1/(λ_max_ϵ) has the same physical unit.

(8a) $$N∼E{}_{T}\left(30\right)/\epsilon$$

or

(8b) $$E{}_{T}\left(30\right)∼N\;\cdot \;\epsilon$$

If both, *E*_T_(30) as well as ϵ would depend strictly linearly on *N* and, thereby, on the oscillator strength of the solvent, the ratio would be constant. This is not the case and thus, *E*_T_(30) is also a function of *N*. In fact, to a first approximation, it seems that *E*_T_(30) is also linearly proportional to *N*, as is reasonably shown in Figure 2. This is an important aspect because it shows that *N* and ϵ are oppositely correlated with *E*_T_(30) (cf. also Figures 1 and 2 in Ref. [8a]) which is documented in Figure 2 due to *E*_T_(30) decreasing with increasing refractive index$\;n_{D}^{20}$ . It is worth noting that $n_{D}^{20}$ and *ϵ* are naturally coupled according to the Kramers‐Kronig relation, thus a linear increase of ϵ results in a linear increase of $n_{D}^{20}$ .^[8c]^ This clear result of Figure 2 makes the interpretation of *E*_T_(30) as a function of *N* and/or ϵ in terms of dipolar versus polarizability effects challenging.

**Figure 3:**
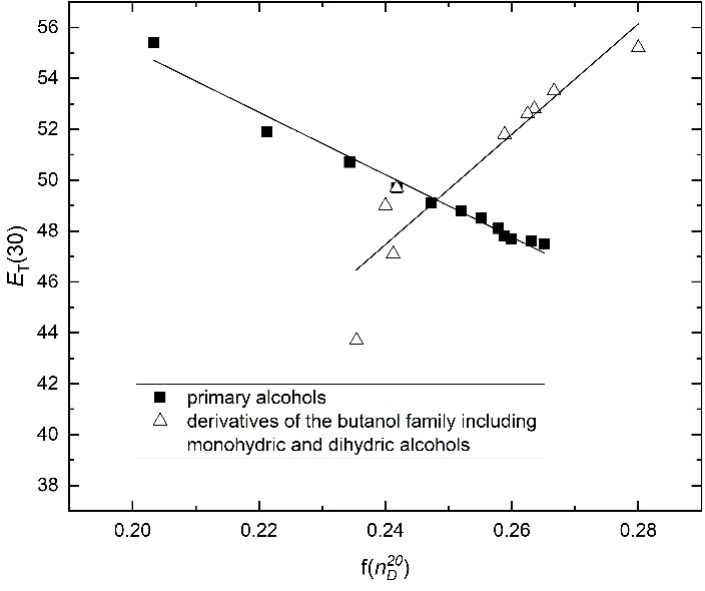
Separate correlations of *E*_T_(30) as a function of f($n_{D}^{20}$ ) for 12 primary alcohols C_1_ to C_12_ (black squares) and nine derivatives of the butanol family including 4 monohydric, 4 dihydric alcohols and 1,2,4‐butanetriol (open triangles).

With respect to c), dihydric and polyhydric alcohols as well as amino alcohols have greater *E*_T_(30) values than expected from Equation (5). They are clearly located above the relationship of the primary ones. If *E*_T_(30) is plotted as a function of f($n_{D}^{20}$ )=[($n_{D}^{20}$ )^2^−1]/[($n_{D}^{20}$ )^2^+2] for primary alcohols and monohydric and dihydric alcohols of the butanol family, two opposite separate linear correlations [Equations (9) and (10)] can be recognized as seen in Figure 3. For the butanol family, there is a good correlation of *E*_T_(30) with the refractive index $n_{D}^{20}\;$ including monohydric and dihydric alcohols as can be seen in Figure 3 (open triangles).

(9) $$E{}_{T}\left(30\right)=-66.1\;\cdot \;f\left(n_{D}^{20}\right)+142.5n=11\;(\mathrm{primary}\;\mathrm{alcohols}),\;r=0.988$$

(10) $$E{}_{T}\left(30\right)=216.8\;f\left(n_{D}^{20}\right)-4.56n=9\;(\mathrm{butanol}\;\mathrm{family}),\;r=0.855$$

The slopes with opposite signs for *E*_T_(30) as a function of f*(* $n_{D}^{20}$ ) for primary alcohols from C_1_ to C_12_ and the butanol family [Eqs. (9) and (10)] underline the different physical effects of the refractivity of the C−H bonds compared to the C−O−H bonds on *E*_T_(30) (more accurately, of the changes in the UV spectra due to additional CH_2_ groups compared to OH groups and the corresponding changes of the refractive indices).

The special role of the dihydric alcohols compared to the monohydric ones in terms of their correlation of *E*_T_(30) with $n_{D}^{20}$ shows the special role of the hydrogen bonding network of di‐ and polyhydric alcohols in terms of their polarizability. The dependencies of *E*_T_(30) and f($n_{D}^{20}$ ) (blue) as a function of *N* for various dihydric and polyhydric alcohol families are also opposite, as shown in Figure S3b in the Supporting Information, which is consistent with the result of Figure 3. A straightforward explanation in terms of electromagnetic coupling is not possible in this case. That corresponds to the conclusion of Equations (8a) and (8b) as mentioned before. As already stated, not only the $n_{D}^{20}$ value is of importance but also the dispersion of the refractive index in the range of the *E*_T_(30) values. The monohydric and dihydric (including 1,2,4‐butanetriol) alcohols of the butanol family show separate correlations *E*_T_(30) as function of f($n_{D}^{20}$ ). In this case, the van der Waals forces resulting from the intermolecular interactions due to the hydrogen bond network seem to dominate over the electromagnetic coupling effects. However, there is also a reasonable complementary explanation (see later section on ^129^Xe NMR).

Usually, the influence of the polarizability of the solute and/or the electromagnetic coupling of the solvent to the solute is routinely investigated by testing correlations of a measure with the refractive index $n_{D}^{20}$ according to the Lorentz‐Lorenz equation (see Equation (S1), Supporting Information)[^10^, ^15^, ^16^, ^19d^] According to Linder,^[16]^ the refractive index $n_{D}^{20}\;$ reflects only the dispersive part of the polarizability. These assumptions neglect the fact that the refractive index is mainly an integral manifestation of absorption in a material, that is, the oscillator strengths (cf. Kramers‐Kronig relations).^[8c]^

With respect to finding d) – the fluorinated alcohols TFE and HFIP deviating significantly from the other values – the measured *E*_T_(30) values of these alcohols are clearly greater than the calculated ones. A potential explanation of this behaviour could be that the replacement of hydrogen by fluorine atoms leads to a blueshift of the n→π* transition of the hydroxyl group,^[42]^ which in turn leads to a decrease of the dispersion of the refractive index function around the *E*_T_(30) value. On the other hand, the fluorinated alcohols have a comparably high density, which also has to be factored in, like the lower number of C−H bonds and the related lower oscillator strengths of the C−H transitions [see also the comment after Equation (12)].

However, monohydric amino alcohols and alkoxy alcohols approach the straight line *E*_T_(30) versus *N* for the primary representatives.

We now use the impressively good correlation of the primary alcohols [Eq. (5)] as a reference relationship to recognize why deviations from *E*_T_(30) occur compared to other alcohol derivatives. Measured *E*_T_(30) values are taken from Refs. [5,43] and compiled in Table S1.

It is assumed that the effective *D*_HBD_ values for secondary and tertiary alcohols are lower than for the primary alcohols by a certain factor because of their different liquid structures.[^1^, ^3^, ^23^, ^24^, ^25^] It is predicted that a hypothetical factor *f*, that determines the efficiency of OH dipolar group action, depends on their position along the alkyl chain according to Ref. [1] We have assumed that this is the case and simply calculated the factor *f* for several monohydric secondary and tertiary as well as polyhydric alcohols from the deviation of the excellent linear relationship given by Equation (5). The weighting factor *f* is then calculated by Equation (11). It is the ratio of the actually measured to the theoretically calculated *E_T_* (30) value using Equation (5). Thus, *f*=1 is automatically set for primary alcohols.

(11) $$f=E{}_{T}\left(\mathrm{measured}\right)/E{}_{T}\left(\mathrm{theoretically}\;\mathrm{calculated}\;\mathrm{by}\;\mathrm{Eq}.\;\left(5\right)\right)$$

The so‐determined *f* factors for a series of alcohols are shown in Table S1. As stated before, the *E*_T_(30) value (calculated) from Equation (5) for secondary and tertiary alcohols is always greater than the actually measured value. From the results of the *f* data (Table S1) it is clear that the *E*_T_(30) polarity of secondary alcohols is only about 94–96 % compared to a primary alcohol. For tertiary alcohols, it is about 79 % (3‐ethyl‐2,4‐dimethyl‐3‐pentanol) to 87 % (^*t*^BuOH) compared to a primary alcohol. The more the OH group is shielded by alkyl substituents, the smaller *f* is, which seems reasonable. An alternative reasoning would be that the smaller density of secondary and tertiary alcohols leads to a decrease of oscillator strength and, thereby, to lower *E*_T_(30) values.

**Figure 4:**
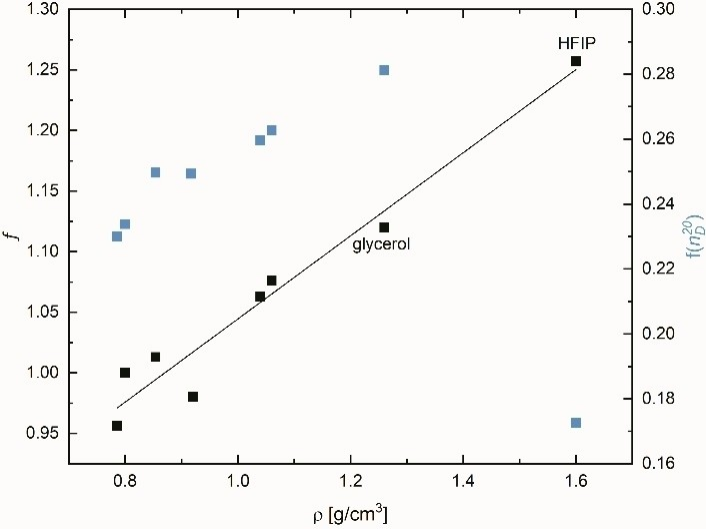
Correlation of the weighting factor *f* and f($n_{D}^{20}$ ), respectively, as a function of density for the C_3_ alcohol family; HFIP is excluded for the correlation with f($n_{D}^{20}$ ).

Therefore, the *f* factor can be clearly physically reasoned. The calculated *f* factor significantly correlates with the density ρ of the respective solvent. The larger the density, the greater *f* (see Figure 4 and Equation (12) for the propanol family and Figures S4–S7, Supporting Information, for the other alcohol families).

(12) f=0.343·ρ+0.701n=8,r=0.974,sd=0.022

It should be noted that HFIP fits well into the correlation *f* as a function of density, but not into the relationship *f* as a function of f($n_{D}^{20}$ ).

The actually lower refractive index values $n_{D}^{20}$ for TFE and HFIP are clearly due to the lower atom polarization and, as a consequence, lower oscillator strengths, of the C−F bond (atomic refraction of F=0.95) compared to a C−H bond (atomic refraction of H=1.1).^[44]^ For this reason, despite the high density ρ, the refractive index is lower and no longer fits into the linear correlation of the refractive index $n_{D}^{20}$ as a function of ρ (see Figure 4). To discuss this point in more details, far‐UV spectra of fluorinated alcohols would be helpful to see how the replacement of H by F changes the spectra and oscillator strengths. The low $n_{D}^{20}$ values of fluorinated alcohols are nevertheless a strong indicator that overall oscillator strengths decrease. Hence, these two solvents and other fluorinated ones must be considered separately.

Therefore, the too strong decrease in *E*_T_(30) of sterically hindered alcohols like ^*t*^BuOH, EDMP or DMP with respect to *N* cannot be explained by the influence of the lower density of these solvents. The deviations of these alcohols are determined by the interaction mechanism of the respective alcohol with **B30** or these deviations are determined by the lower refractive indices/lower oscillator strengths of these solvents as indicated in Figure 2.

The crucial factor on *E*_T_(30) is the steric hindrance in the interaction of **B30** with tertiary alcohols^[5e]^ and/or the decrease of oscillator strengths and associated electromagnetic coupling.^[8a]^

To integrate both the number of OH groups and their effectiveness into the *D*_HBD_ concept,^[21]^ we combined the idea of Equation (2) with the results from Figure 4 and Table S1.

The additivity of the OH groups and their position can be theoretically calculated using the solvents’ molar concentration *N* and the sum of OH groups with the determined impact factor *f*_s_ and *f*_t_ of the OH group position as proposed by Equation (13). *D*_HBD_ can therefore be calculated theoretically with the aid of Equation 13:

(13) $$D{}_{\mathrm{HBD}}=N\;\cdot \;\left(f{}_{p}n{}_{p}+f{}_{s}n{}_{s}+f{}_{t}n{}_{t}\right)=N\;\cdot \;\Sigma n$$

with n_p_ ‐ number of primary OH groups, n_s_ ‐ number of secondary OH groups, and n_t_ ‐ number of tertiary OH groups within the molecule. The *f*_s_ and *f*_t_ factors, respectively, are derived from the *f* values of corresponding monohydric secondary and tertiary alcohols as references, as shown in Table S1 (Supporting Information). So, for glycerol, the actual Σ*n* would be 2.956, since Σ*n*=2 ⋅ 1+1 ⋅ 0.956, given the position factors for two primary OH groups and one secondary OH group from 2‐propanol. The calculated *D*_HBD_ data for secondary and tertiary alcohols are presented in Table S2.

Whether Equation (13) provides a fundamentally relevant statement is proven by correlations of Σ*n* with physical constants of the alcohol derivatives. As expected from Figure 4, the physical density ρ also plays a dominant role here. The density ρ was used as a benchmark in combination with the refractive index $n_{D}^{20}$ , since both quantities are naturally linked by the Lorentz‐Lorenz equation [see Equation (S1)]. Thus, for the test correlations, polyols for which no *E*_T_(30) value is known can also be used as reference compounds. The densities of different polyols, solid erythritol and solid xylitol, and the Σ*n* values theoretically determined from Equation (13) were also included in the correlations. Experimentally determined density data and refractive index data $n_{D}^{20}\;$ of various alcohols are collected in Table S3 (Supporting Information).

Equation (13) is scientifically reasonable because there are highly significant correlations of Σ*n* and ρ or $n_{D}^{20}\;$ for the alcohol families C_3_ to C_8_ (see Figures S13–S15).

**Figure 5:**
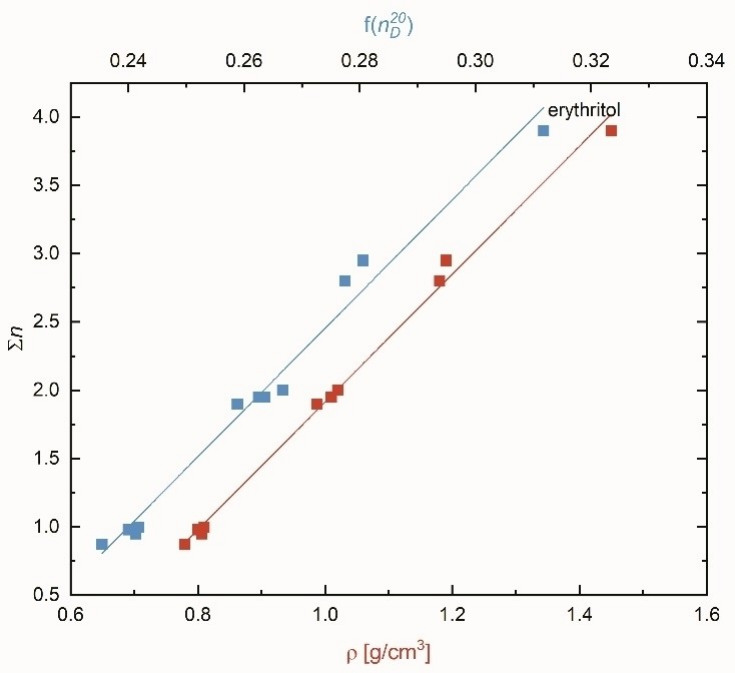
Correlations of Σ*n* as a function of density and refractive index function for the butanol family, including the solid erythritol.

The results are therefore impressive, because the Σ*n* parameters of mono‐ and polyhydric propanol and butanol derivatives, calculated by using Equation (13), correlate linearly with their densities ρ, regardless of whether the compound is liquid or solid at 20–25 °C; see Figure 5 and Equations (14)–(16).

As can be seen from Equations (14) and (15a), correlation qualities for the propanol and butanol families are excellent, (Figure S5, Supporting Information), because the calculated *f* of the secondary OH group integrated in the Σ*n* value of polyhydric alcohols fits exactly.

(14) Σn=4.13·ρ-2.3n=5(propanolderivatives),r=0.998

(15a) Σn=4.678·ρ-2.7654n=11(butanolderivatives),r=0.997

These results also hold for the correlation of the density with the refractive index for a series of alcohol families (for the respective data, see Table S3, Supporting Information). A linear correlation of any parameter with the physical density inevitably indicates that refraction is also coupled with it (see Figure 2) according to the established Gladstone‐Dale relationship,^[45]^ which can be seen as an approximation derived from the Lorentz‐Lorenz relation (excluding fluorinated solvents).^[41]^ The complementary correlation of Σ*n* as a function of f($n_{D}^{20}$ ) for the butanol family is also shown in Figure 5 and Equation 15b).

(15b) $$\Sigma n=21.98\;\cdot \;f(n_{D}^{20})-29.66n=11\;(\mathrm{butanol}\;\mathrm{family}),\;r=0.990$$

In particular, the correct order of the gradual decrease of *E*_T_(30) from 1,2,4‐butanetriol>1,4‐butanediol>1,3‐butanediol>1,2‐butanediol>2,3‐butanediol with respect to Σ*n* is convincing. This is also a good proof for the reliability of the *f*_s_ factors for secondary propanol and butanol derivatives.

However, the situation with alcohols with more than four carbon atoms is more complicated, regardless of whether they are monohydric or polyhydric alcohols. Thus, the classification *f* from Equation (11) for the C_5_, C_6_ and C_7_ alcohol families is not unambiguous. The reason behind this lies in the density of these alcohol families (data collection see Table S3) no longer strictly following the sequence primary>secondary>tertiary alcohol.^[1]^ Therefore, correlation qualities Σ*n* as function of ρ for C_5_, C_6_ and C_7_ alcohol families are slightly worsened [for an example, see Equation 16].

(16) Σn=5.888·ρ-3.777n=14(pentanolfamily),r=0.982

As a consequence, “position factors” relating to *f* are not reasonably calculable for C_5_, C_6_ and C_7_ alcohol families. Nevertheless, the densities of these alcohol families significantly correlate with the corresponding refractive index ($n_{D}^{20}$ ) according to the Gladstone‐Dale relation (see data of Table S3).^[45]^ Overall, the correlations of Σ*n* as a function of density for the alcohol derivatives, shown in Figures S8–S11, are conclusive. Therefore, the Σ*n* value is not a pure factor reflecting the OH position, but is also influenced by the electromagnetic coupling of the oscillators of the solute and of the alcohol derivative. Thus, the slope (ΔΣ/Δρ) of each correlation is preferentially determined by the number of carbon atoms of the alcohol family (Figure S12)

Importantly, the higher *E*_T_(30) values of polyhydric alcohols, TFE and HFIP compared to primary alcohols with respect to *N* are clearly due to the influence of density, but in different ways for both groups of solvents. The good integration of TFE and HFIP in the linear *f* factor density correlations is particularly worth highlighting (see Figures S5 and S6, Supporting Information). Consequently, hypothetical Σ*n* values for TFE and HFIP are calculable from the density correlation (see also the footnotes to the caption of Figure S13a). These theoretically proposed Σ*n* values for TFE and HFIP would be approximately 3 and 4, respectively. They are in complete agreement with actual demanding physical measurements for these solvents.[^46^, ^47^, ^48^, ^49^, ^50^, ^51^] Thus, for TFE, it is established that clusters of three molecules are present at 25 °C, but no extended polymeric hydrogen bond network is operative.^[46]^ For HFIP, the situation is rather complicated from a scientific point of view.[^47^, ^48^, ^49^, ^50^, ^51^] The problem is that HFIP is not really homogeneous and exhibits a nano‐heterogeneity as a function of the specificity of the solute, so that the true solvent‐cluster size can locally vary quite significantly. HFIP can form a zig‐zag structure, helices or cyclic domains with up to six HFIP molecules, depending on its partner.[^49^, ^50^] Consequently, the actual domain size is determined by the interaction between HFIP and its solutes. Thus, Σ*n* can vary as a function of the component to be solvated as explained for dye **33** (Figure 1) in the Supporting Information. This is certainly true for the solvation of solvatochromic dyes; a different nano‐heterogeneity is formed for each, which also explains the variations in the determination of the *E_T_* (30*)* value in the literature (see Tables S1, S2 and S4 as well as references in the Supporting Information).[^5a^, ^43a^] Therefore, it is fundamentally questionable to set a fixed solvent parameter for HFIP if it has been determined by any dissolved probe molecules.

Hence, in the following, the effective OH group density *D*_HBD_ from Equation (13) will be used for correlation with the measurand EP according to Equation (17) (Data see Table S2, Supporting Information).

(17) $$EP=a\;\cdot \;D{}_{\mathrm{HBD}}+b$$

However, it should be noted that the factor *f* is derived from the solvatochromism of the standard dye **B30** in primary alcohols as reference. It is conceivable that the effective OH density for secondary and tertiary alcohols also depends on the size and shape of the probe molecule to be solvated. Consequently, when evaluating other solvatochromic polarity probes or solutes, for example Xe or the Y‐scale based on ^*t*^BuCl, special attention should be paid to the deviations for secondary and tertiary alcohols.

The results of this section as well as those shown in the corresponding part of the Supporting Information illustrate the importance of the double function of the alcohol density, since both *N* and Σ*n* are determined independently of it. The double function is attributed to the fact that Σ *n* is naturally coupled to the refractive index of the solvent molecule (see also Figure 5).[^10^, ^45^]

### *E*_T_(30) as function of the refined *D*_HBD_ parameter

**Figure 6:**
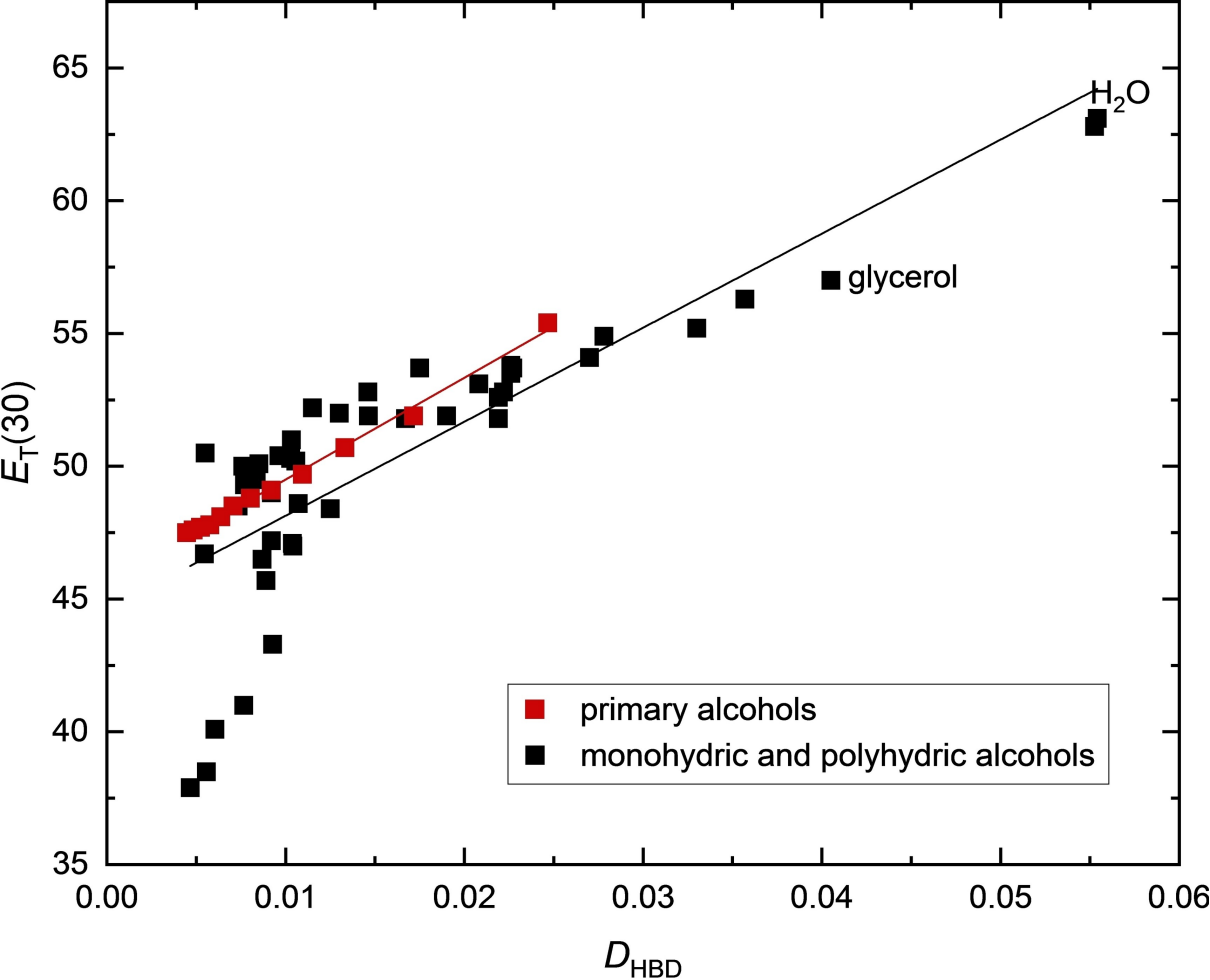
Plot of *E*_T_(30) as a function of *D*_HBD_ for 54 HBD solvents with p*K*_a_>14, including monohydric and dihydric alcohols, glycerol and 1,2,4‐butanetriol, D_2_O and water. Primary *n*‐alcohols are represented by red squares.

To check the usefulness of the calculated *D*_HBD_ parameters, the correlation of *E*_T_(30) with *D*_HBD_ was tested. Results are shown in Figure 6 and Equations (18a) and (18b).

The sound classification of seven dihydric and the two trihydric alcohols glycerol and 1,2,4‐butanetriol proves the appropriateness of the *D*_HBD_ concept. Equation (18a) holds for primary and secondary alcohols, polyols and amino alcohols.

(18a) $$E{}_{T}\left(30\right)=306.07\;\cdot \;D{}_{\mathrm{HBD}}+46.423n=43\;(\mathrm{all}\;\mathrm{solvents},\;\mathrm{excluding}\;\mathrm{tertiary}\;\mathrm{alcohols},\mathrm{DMP}\;\mathrm{and}\;\mathrm{NH}\text{-}\mathrm{acidic}\;\mathrm{solvents}),r=0.9741$$

The special role of the tertiary and steric shielding alcohols is also demonstrated in Figure 6 in the range of *E*_T_(30) from 37 to 43 kcal mol^−1^. Considering the current argument that the dye/solvent complex is the actual species responsible for the solvatochromic shift,^[9]^ the deviations of the tertiary alcohols and DMP can also be easily explained.

Di‐ and trihydric alcohols, like water, can form a three‐dimensional network of hydrogen bonds. It is noticeable that the correlation line of these di‐ and trihydric alcohols, Equation (18b), runs parallel and with a significant lower slope to the primary alcohols [red line in Figure 6 and Equation (5); regarding the special role of triethylene glycol, see Equation 20].

(18b) $$E{}_{T}\left(30\right)=262\;\cdot \;D{}_{\mathrm{HBD}}+47.7n=14\;(\mathrm{including}\;\mathrm{diols},\;\mathrm{triols}\;\mathrm{and}\;\mathrm{water},\mathrm{excluding}\;\mathrm{triethylene}\;\mathrm{glycol}),r=0.985$$

Thus, despite the larger refractive index $n_{D}^{20}$ of the polyhydric alcohols, the *E*_T_(30) value is smaller which would be consistent with the interpretation of Figure 3 that the (intermolecular) hydrogen bond network structure of the polyhydric alcohol certainly also plays a role. Water always fits better within the group of polyhydric alcohols than with the primary alcohols [compare Equation (4) with Equation (5)].

Hence, Figure 6 shows that a detailed analysis of *E*_T_(30) values as a function of *D*_HBD_ is now possible for various HBD solvent groups. A more comprehensive study including sugar and phenol derivatives^[52]^ will be presented in a subsequent publication and is not covered in this paper for reasons of focus.^[53]^

The correlation analysis according to Equation (17) can also be applied to other solvent parameters.[^5^, ^18^, ^19^, ^43^, ^54^, ^55^, ^56^, ^57^] A further study will also investigate whether non‐alcoholic HBD solvents such as formamide, *N*‐methylformamide, *N*‐methylacetamide, aniline and *N*‐methylaniline can be treated with the *D*_HBD_ model. From the electromagnetic coupling point of view, this approach is justified as long as the refractive index and its dispersion, which represent the integral oscillator strengths and the band positions, do not change noticeably. *E*_T_(30) values should also not change as long as it is the electromagnetic coupling that strongly influences the solvatochromism.

### Correlation of ^129^Xe NMR shift data with *D*_HBD_

**Figure 7:**
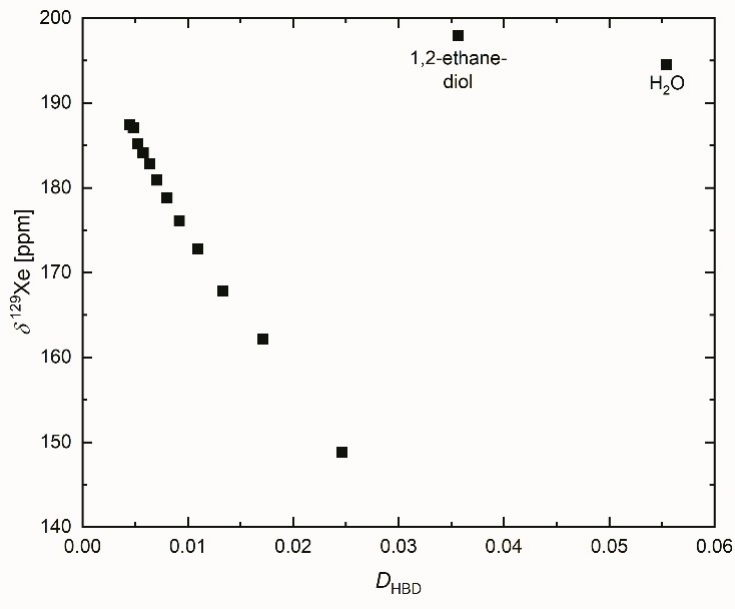
Correlation of the ^129^Xe NMR chemical shift as a function of *D*_HBD_ for primary alcohols, 1,2‐ethanediol and water.

The results from literature are a clear indication that the chemical shift of Xe in common organic solvents is attributed to physically determined van der Waals interactions.[^27^, ^58^, ^59^, ^60^, ^61^] For this reason, the chemical shift δ^129^Xe of Xe dissolved in primary alcohols increases with increasing the sum of number of carbon atoms and oxygen atoms.[^58^, ^60^] Accordingly, δ^129^Xe increases linearly with decreasing *N*=*D*_HBD_ for primary alcohols because in the same order the number of constituting atoms in the alcohol increases. (see Figure 7). However, water and 1,2‐ethandiol do not fit in the relationship for primary alcohols. According to the plot for primary alcohols in Figure 7, a hypothetical chemical shift of about 75 ppm would be expected for water. The measured δ^129^Xe, however, is 196 ppm.[^27^, ^60^, ^61^]

The special classification of water and 1,2‐ethandiol is profoundly interpreted in the significant study by Lim at al.^[58]^ The authors convincingly argued that both solvents show a great van der Waals energy contribution due to the hydrogen bond network compared to primary ones. Primary alcohols only form a two‐dimensional network.[^1^, ^3^] The comparison of the ^129^Xe NMR shift with the refractive index for water shows that Xe “feels” (recognizes) a stronger van der Waals contribution of water as expressed by $n_{D}^{20}$ which is a clear indication of different coupling of the oscillator of solvent and solute for monohydric and polyhydric alcohols.[^8^, ^41^] This particular result for water is very important because it shows the unique role of the water and 1,2‐ethandiol bulk due to the special role of the three‐dimensional hydrogen bond network on physically van der Waals force‐determined properties. Xe dissolved in water behaves completely different compared to solutions with other solvents, because of the unusual T‐dependence of the ^129^Xe NMR chemical shift.^[60]^

Due to the lack of literature data on ^129^Xe NMR measurements in glycerol, various diols or secondary and tertiary alcohols, one has to evaluate complementary literature data from literature related to the problem as presented and discussed in Chapter 2.7.1 in the Supporting Information (^129^Xe NMR Data discussion)..^[61]^

This literature review shows that the strong van der Waals forces are due to the complexity of the hydrogen bond network. However, one has to distinguish between the van der Waals forces resulting from the (three‐dimensional) hydrogen bond network and those of the alkyl chain or the aromatic substituents of the alcohols. This is the crucial point. The different qualitative influence of alkyl chains compared to the three‐dimensional hydrogen bond network with respect to the *van der Waals* interaction probably also explains the result of Figure 3 and Figure S3b (Supporting Information).

### Correlation of lg*k*_1_ with *D*_HBD_

Since the fundamental work of Grunewald and Winstein,^[28]^ the solvent‐dependent rate constants (lg*k*_1_) of the s_n_ 1 solvolysis reaction of ^*t*^BuCl with water (Scheme 1) have become established as suitable criteria for classifying the versatile polarity effects of solvents.[^5d^, ^28^, ^32^, ^33^, ^34^, ^62^, ^63^, ^64^, ^65^, ^66^, ^67^] There are also well established correlations between *Y* or lg*k*_1_ and the *E*_T_(30) values in the literature, with variations of certain solvent classes.[^11b^, ^32^] This is an indication that influences of solvent on rates of reactions are also attributed to coupling of the transition state with the oscillating of the solvent.

**Figure 8:**
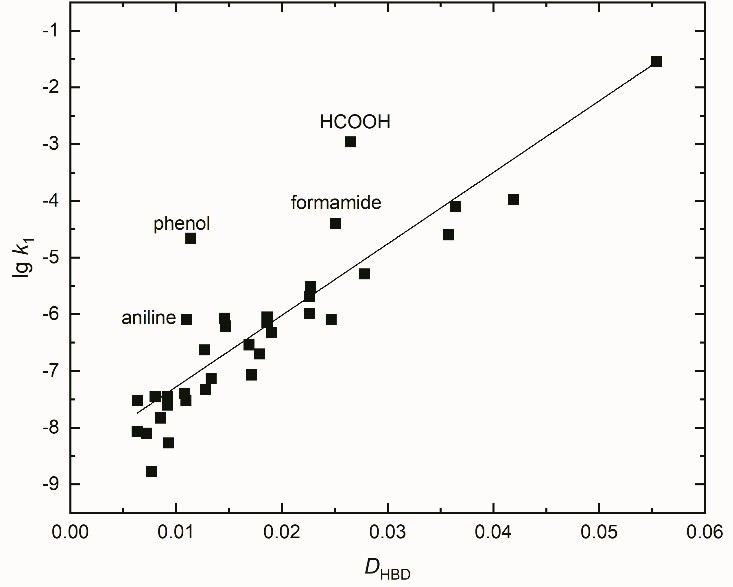
Correlation of lg*k*_1_ (from column 3 of Table S5) as a function of *D*_HBD_ for pure HBD solvents including HCOOH, aniline, phenol, and formamide.

Winstein's *Y* parameter correlates excellently with *D*_HBD_ for six pure alcoholic solvents including acetic acid and water, as shown in the Supporting Information [see Figure S16 and Equation (S21a)]. However, to analyse the whole body of literature on this field is hardly manageable (see Refs. [5d, 11b, 28, 32–34, 62–67] and citations). A compilation of lg*k*_1_ data used for this study is given in Table S5 (Supporting Information) with references these data have been taken from as indicated. Most of the data comes from the comprehensive work of Dvorko.^[34]^ It is essential to explain both the data itself and the use of those from different authors (see also footnote comment to Table S5). At first, we have exclusively correlated the lg*k*_1_ from Ref. [34] as function of *D*_HBD_, including phenol, formic acid, aniline and formamide, assuming *D*_HBD_=*N* for non‐alcoholic solvents (see Figure 8).

(19a) $$\mathrm{lg}k{}_{1}=126.1\;\cdot \;D{}_{\mathrm{HBD}}-8.542n=36,\;r=0.877$$

It is noticeable that formic acid deviates from the straight line due to its greater acidity (p*K*a=3.7). Phenol and aniline are aromatic solvents that also clearly show higher lg*k*_1_ values than expected, similar to formamide. In recent literature, phenol and HCOOH data have always been completely omitted by the authors in correlation analyses.[^64^, ^65^, ^66^]

If the four deviants HCOOH, aniline, phenol, and formamide are omitted, the correlation is improved significantly [Eq. 19b)].

(19b) $$\mathrm{lg}k{}_{1}=125.5\;\cdot \;D{}_{\mathrm{HBD}}-8.75n=32,\;r=0.97$$

Interestingly, for the aromatic alcohol benzyl alcohol, lg*k*_1_ values for ^*t*^BuCl are not reported.[^34^, ^64^, ^65^, ^66^] However, for the hydrolysis of 1‐bromo‐1‐methylcyclopentane and related substrates, benzyl alcohol was sometimes used as solvent.^[34]^ Altogether, these lg*k*_1_ values measured in benzyl alcohol do also not fit in any reasonable correlation with *N* or *D*_HBD_, being too high in relation to its *D*_HBD_ value. This finding is consistent with the results that lg*k*_1_ values measured in aniline or phenol are too high in relation to *D*_HBD_. The explanation seems simple: the *D*_HBD_ quantity is not appropriate to describe polarizability effects of π‐electron systems of solvents upon a solute. Also, it does not represent conventional acidity as the point for HCOOH shows. From this, it can be concluded that both strongly acidic solvents in terms of p*K*_a_ and strongly polarisable solvents exert a disproportionately accelerating effect on the solvolysis reaction.

The previous literature doctrine of the solvent influence on lg*k*_1_ is that both solvent acidity in terms of HBD strength (about 60 %) and dipolarity/polarizability of the solvent (about 40 %) accelerate the reaction, which would apparently be consistent with this.[^32^, ^64^, ^66^] However, this is not really the case because the empirical polarity scales used for HBD solvents do not reflect true acid H‐bond donor scales but the dipolarity of the hydrogen bond network, as shown in this paper. This is why the authors omitted all HCOOH and phenol data from correlation analyses, because it permanently disturbs their explanation.[^64^, ^65^, ^66^] The lg*k*_1_ data measured in benzyl alcohol would also strongly interfere.^[34]^

Due to the fact that the Catalan SB parameters for alcohols do not reflect basicity,[^13b^, ^19b^] these results of the correlation analyses cannot really be taken seriously.^[66]^ Furthermore, the validity of the conclusions of the recent papers[^64^, ^65^, ^66^] is therefore difficult to assess because everyone has omitted in detail few solvents that they consider unsuitable. Thus, formic acid, 1,4‐dioxane, acetic acid, and phenol are often omitted, but no justifications as to why this is the case are given.^[65]^

However, the earlier analysis of lg*k*_1_ in Ref. [67] which includes the combination of *E*_T_(30) and refractive index $n_{D}^{20}$ for explaining lg*k*_1_ is significant. Thus, this lowering of the transition state (TS) of the ^*t*^BuCl substrate in the solvolysis reaction is essentially dependent on the global polarity and not on specific interactions. The excellent fitting of water within this correlation should be emphasized.

Importantly, the detailed mechanism of ^*t*^BuCl solvolysis is also determined by the nature of the solvent as shown by the strong dependence of the activation entropy Δ*S* ^≠^ on solvent structure.[^28c^, ^62^] In water and in the gas phase, the activation entropy is positive. Obviously, the initial dissociation step from ^*t*^BuCl to the [^*t*^Bu^+^Cl^−^]* is Δ*S* ^≠^‐determining in water and not the solvation of this state. Accordingly, the high global polarity of water is sufficient to achieve the activation of the substrate. In all other solvents, the activation entropy is negative, proving evident interaction of the suggested [^*t*^Bu^+^Cl^−^]* TS with the solvent. Especially for phenol and aniline, a strong negative activation entropy (Δ*S* ^≠^≈−100 J mol^−1^ K^−1^) is measured. This result also holds for other (aprotic) aromatic solvents like acetophenone (Δ*S* ^≠^≈−164 J mol^−1^ K^−1^) or benzonitrile. Accordingly, the aromatic HBD solvents do not fit in the correlation of lg*k*_1_ with *D*_HBD_ (Figure 8). Phenol (ϵ_r_=8) and aniline (ϵ_r_=7) have low static dielectric constants, but a high polarizability. This deviation would also hold for benzyl alcohol derived by comparison with related kinetic data from the literature.

**Figure 9:**
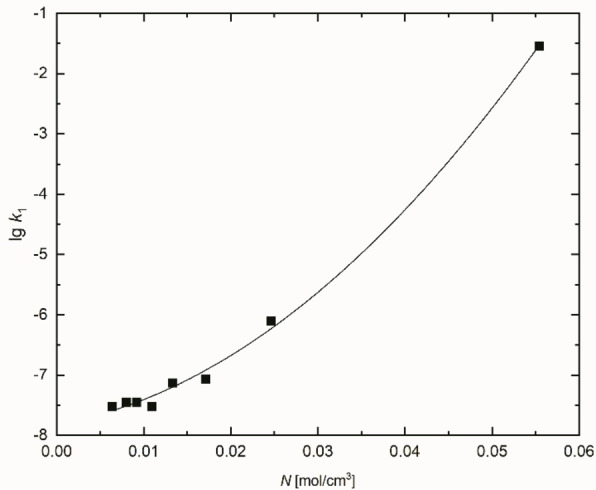
Plotting of lg *k*_1_ for ^*t*^BuCl as a polynomic function versus *N*=*D*_HBD_ for primary alcohols and water; this curve is hidden in Figure 8.

In contrast, in (stronger) protic nonaromatic solvents (formic acid, acetic acid, methanol, ethanol, formamide) with high *D*_HBD_, the activation entropy is less negative (−20>Δ*S* ^≠^>−7). These results are a clear indication of the change in the balance of solvation forces between the solvation of the “transition state” of the solvent shell with that of the global volume. The stronger the interaction of the ^*t*^BuCl TS with solvents in the first solvation shell, the more negative the activation entropy. Long‐chain alcohols (*n*‐hexanol, *n*‐octanol) and branched alcohols (*tert*‐butanol) show Δ*S* ^≠^ of about −70 J mol^−1^ K^−1^ indicating stronger dispersion interaction with the TS in accordance with Ref. [67]. The activation entropy data from Ref. [62] raise the fundamental concern that single linear free energy relationship (LFER) should not be routinely used to elucidate the influence of solvents on lg*k*_1_. Within the reaction series investigated in Table S5 (Supporting Information), the Δ*S* ^≠^ changes drastically, indicating qualitatively very different solvation structures of the transition state depending on the solvent family. The concept of LFER requires that the entropy changes within a reaction series should not be too large. For this reason, a linear correlation of lg*k*_1_ with *D*_HBD_ or any other parameter is not to be expected since solvents with highly polarizable groups are included. This concern is strongly supported by the non‐linear plot of lg*k*_1_ as a function of *N* for the homologous series of primary alcohols, including water (see Figure 9).

The linearity od lg*k*_1_ as a function of *N* only holds for water, methanol and ethanol, but there are no major differences in lg*k*_1_ for 1‐butanol, 1‐pentanol, 1‐hexanol and 1‐octanol although *N* systematically decreases with increasing *n*‐alkyl chain length. If the *D*_HBD_ quantity (or *N*) would be the only factor for influencing the lg*k*_1_, a much lower rate constant for long‐chain alcohols would be expected. As expected from the clear linear correlation of *E*_T_(30) with *N* [Eq. (5)], a similar result is obtained when lg*k*_1_ is plotted as a function of *E*_T_(30) for primary alcohols.

Obviously, the energy part of the van der Waals interaction caused by the alkyl groups of the alcohols with the [^*t*^Bu^+^Cl^−^]* TS is not reflected in *N*=*D*_HBD_ or *E*_T_(30), but in Ig*k*_1_. If the polarizability of the alkyl chain would have the dominant impact, a linear correlation of lg*k*_1_ with the molar volume *V*_m_ is expected. This is not observed; plotting lg*k*_1_ as a function of *V*_m_ results in a similar curve compared to Figure 9. There is no differentiation in lg*k*_1_ as function of *V*_m_ in the range from ethanol to 1‐octanol. This is a clear indication that both the dipolarity and the polarizability of alcohol act together. This consideration is supported by the study in Ref. [67] which shows that lg*k*_1_ is a function of both *E*_T_(30) and refractive index $n_{D}^{20}.$ As an additional note to this fact, the introduction of lipophilic alkyl groups on the **B30** dye promotes solubility in non‐polar solvents, but has no effect on the value of the *E*_T_(30) parameter.^[5a]^ This indicates that the electromagnetic coupling of the alcoholic solvent can only be detected with the chromophoric part of **B30** and not with the (alkyl) substituents in the periphery. Similarly, long alkyl groups of alcoholic solvents have no direct effect on the chromophore moiety of **B30**. The influence is only indirectly detected by the decrease of the OH group density because longer alkyl chains lead to on average weaker dipole‐dipole interactions due to the on average larger distances between the OH groups. Pure alcohols can then be described as binary mixtures of the polar OH function with the less polar alkyl chains. This interpretation was already proposed by Langhals, who regarded alcohols as a solvent mixture of OH and alkyl groups.^[11a]^

Thus, the result of Figure 9 is indirect but clear evidence that the dispersion contributions of long‐chain alcohols have a lowering resp. levelling influence on the transition state (accelerating effect) similar to aromatic solvents. Unfortunately, there are too few kinetic data for other s_n_ 1‐active substrates in long alkyl chain alcohols available. However, this result would be consistent with lg*k*_1_ data measured in aniline, benzyl alcohol and phenol.^[34]^

The meaningful kinetic data from Ref. [67] were also used to test the suitability of the *D*_HBD_ parameters for dihydric alcohols. Kinetic data and physical parameters are given in Table S6. This consideration serves, on one hand, to confirm the *D*_HBD_ values for polyhydric alcohols and, on the other hand, to check whether changes of dispersion contributions of polyols do play a role on lg*k*_1_. However, an excellent linear correlation of lg*k*_1_ with both *D*_HBD_ and *E*_T_(30) is found (see Equation (20) and Figure S17, Supporting Information) including lg*k*_1_ data measured in glycerol and water from Ref. [34.] Only triethylene glycol shows a too high lg*k*_1_ value in relation to *D*_HBD_, which can readily be attributed to additional dispersion interactions due to the larger number of C and O atoms in relation to OH groups as indicated in Table S6, which is in accord with the discussion of Figure 9 and Equation (20). Therefore, in retrospect, triethylene glycol does also not fit well in the correlation of *E*_T_(30) as a function of *D*_HBD_, see Equation 18b).

(20) $$\mathrm{lg}k{}_{1}=117.713\;D{}_{\mathrm{HBD}}-8.542n=12,\;r=0.967,\;sd=0.340$$

This result is a clear indication that the contribution of the van der Waals energy of the three‐dimensional hydrogen bonding network of the polyhydric alcohols is comprehensively reflected by the *E*_T_(30) value or *D*_HBD_ and confirms the correctness of the approach in Equation (13). This fact clearly supports the consideration that the deviating solvents in the Figure 8 can be attributed to additional dispersion and genuine acid‐base interactions.

The lg*k*_1_ measured in HFIP is located exactly between water and TFE.^[34]^ HFIP was not considered by us in either correlation analysis. It is suggested that the reactivity of the water reactant is significantly affected by the HFIP solvent which makes the situation complicated.^[68]^

Recently, Abraham has questioned the use of UV/Vis‐spectroscopic parameters derived from the Kamlet‐Taft and Catalan equations for the analysis of linear‐free solvation energy (LFSE) relationships.^[65]^ Basically, this criticism is not justified, because the principle of LSER is fulfilled as explained for solvatochromically determined parameters in the introduction and in the Supporting Information. However, other authors[^64^, ^66^] arrive at almost the same physicochemical statements in multiple square correlation analysis as those obtained from the use of the Abraham water partition coefficients.^[69]^ Unfortunately, the measurement of partition coefficients in binary solvent/water systems always involves the influence of the global volume polarity of water assuming that it is the strongest HBD solvent.^[69]^ Water is not a strong HBD solvent in terms of hydrogen bonding effects to a solute. This misjudgement requires a clear correction. However, the author, in the end, proceeds just as arbitrarily to define the parameter for HBD ability. Thus, Abraham's partition coefficients are actually worthless in terms of their interpretation, but beneficial in terms of accurately describing global volume effects of water mixtures.

**Figure 10:**
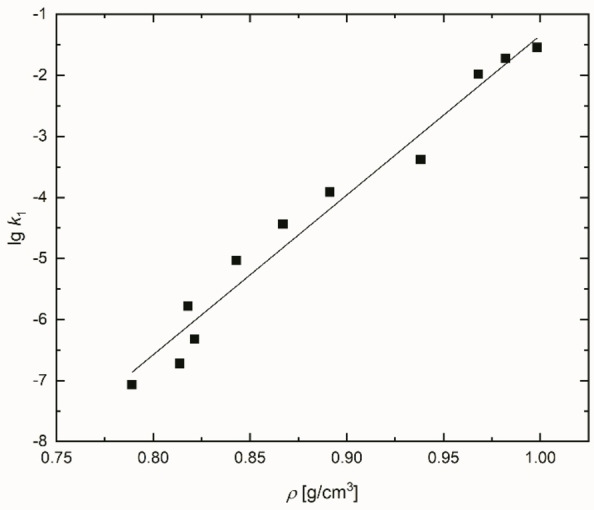
Linear correlation of lg*k*_1_ as a function of density of the ethanol/water mixture. The lg*k*_1_ data are taken from Ref. [66].

This concern is supported by the fact that the lg*k*_1_ of ethanol/water mixtures is nearly a linear function of their density as shown in Figure 10 and Equation (21); for methanol/water mixtures, see Figure S18 and Equation (S24). Density data of water/alcohol mixtures are tabulated in the literature (see also the Supporting Information).

(21) $$\mathrm{lg}k{}_{1}=26.2\;\cdot \;\sigma -27.51n=11\;(\mathrm{ethanol}/\mathrm{water}\;\mathrm{mixture}),\;r=0.988$$

The statement of Figure 10 agrees well with the results of Figure 8 in Ref. [11b] which shows an approximately linear dependence of Δ*G* ^≠^ (free activation energy) on the water/ethanol composition of the solvent. This is substantiated by the fact that composition and density are an approximately linear function for the ethanol/water mixture (see Table S7, Supporting Information). However, the situation is even more complex. It depends on which variables of the solvent mixture are correlated with lg*k*_1_. For this purpose, the average molar concentration *N*_av_ of the sum of acting dipoles *N*_av_ of the mixture is calculated [Eq. 22].

(22) $$N{}_{av}=\rho {}_{m}/M{}_{\mathrm{AV}}=\rho {}_{m}/\left(x{}_{1}M{}_{1}+x{}_{2}M{}_{2}\right)$$

with x_1_ and x_2_ being the mol fraction of solvent 1 and solvent 2, respectively (x_1_+x_2_ =1). *M*_1_ and *M*_2_ are the molar masses of solvent 1 and 2, respectively. To accurately calculate *N*_av_, independently measured knowledge of the density of the solvent mixture ρ_m_ is required.

The usefulness of *N*_av_ is demonstrated by the fact that *E*_T_(30) is an excellent linear function of *N*_av_ of the ethanol/water mixture [Eq. (23); see also Figure S19, Supporting Information] and not a curved (logarithmic) function like *E*_T_(30) as a function of solvent composition.[^5f^, ^11^, ^20^, ^70^] The consequences of results of Equation (22) and (23) for evaluating aqueous solvent mixtures and others will be presented in a following paper.

(23) $$E{}_{T}\left(30\right)=301.55\;\cdot \;N{}_{\mathrm{av}}+47.173n=14,\;r=0.997$$

According to Equation (23), the solvent mixture water/ethanol does not fit the linear dependence of Figure 8, but would linearly fit Figure 6.

Thus, if lg*k*_1_ is plotted as a function of *N_av_* or *E*_T_(30) (see Table S7 and Figure S20, Supporting Information) of the solvent mixture, the result is not a linear, but an asymptotic curve. Even low water concentrations have a strong accelerating effect due to the influence on the activation energy Δ*H* ^≠^ that is decreased with *N*_av_ in this concentration range.[^11b^, ^28c^, ^62^] Furthermore, the overall plot of lg*k*_1_ as a function of *N*_av_ roughly corresponds to the course of Δ*S* ^≠^ as a function of ethanol/water solvent composition.[^11b^, ^28c^] However, these multifaceted aspects of the energetic and entropic factors on lg*k*_1_ (Refs. [11b, 28c]) in relation to *N*_av_ will be analysed in future work.^[53]^

As a result of these considerations, it is even doubtful whether a linear relationship (Figure 8) is meaningful, since the mechanism changes with increasing amounts of water in solvent mixtures or through solvent changes. Despite these concerns, it can be clearly proven that specific HBD properties in terms of energetic contributions (hydrogen bonds) do not at all have an influence on the reactivity of ^*t*^BuCl in aqueous systems. In each case, it is the number of dipoles that correlates with lg*k*_1_, but in different ways. For this reason, almost all conclusions regarding mechanistic aspects from the recent literature are actually doubtful.[^64^, ^65^, ^66^] The result of this section requires a rethinking of the modern interpretation of protic solvent effects in solvolysis reactions.[^64^, ^65^, ^66^] The use of the *D*_HBD_ quantity for the evaluation of the solvolysis reaction is strongly recommended to recognize effects of hydrogen bond network polarity. The quantity *D*_HBD_ or *N* represents the pure OH group density and neither contributions of dispersion nor real acid‐base interactions. For this purpose, the parameter *E*_T_(30) is conditionally suitable if it is used correctly with regard to the global polarity for pure mono‐ and polyhydric alcohols. For solvent mixtures of water and alcohols, its usefulness is still unclear and requires further research due to the influence of entropic factors on lg*k*_1_ in aqueous solvent mixtures.[^11b^, ^28c^, ^62^] However, correlations of lg*k*_1_ with *E*_T_(30)‐related scales such as Kosower's *Z* will be discussed in subsequent contributions due to inconsistencies of *Z* data for solvent mixtures.[^54a^, ^70^]

The *D*_HBD_ application should be used as a heuristic tool to elucidate changes in the reaction mechanism and solvation states. Furthermore, an extension of the *D*_HBD_ concept should be developed for the ionizing power of aprotic solvents taking into account the number of real acting dipoles per volume.^[13b]^ However, as the order of dependence of lg*k*_1_ on the solvent is also co‐determined by the substrate,[^33^, ^71^] especially in the case for special halogenated (highly polarizable) and etheric (less polarizable) solvents, the definition of a general scale for the ionisation power of the solvent becomes questionable.^[71]^ Also, the static dipole moment of ^*t*^BuCl is solvent‐dependent.^[72]^

## Conclusions

The purely physically determined *D*_HBD_ parameter of alcoholic solvents has been proven as a reliable quantity to accurately describe solvent‐dependent processes of various protic solvents. The demonstration of excellent correlations of the UV/Vis absorption energy (band position) of the solvatochromic probe molecules **B30** with the *D*_HBD_ parameter for alcoholic solvents shows the dominance of the solvent volume properties (global polarity) in explaining the solvatochromic UV/Vis shifts. Obviously, the actual solvatochromic species is the **B30**/solvent complex, which was still the missing link in the interpretation.^[9]^

Many previous studies have noted that hydrogen bonds between **B30** and HBD solvents clearly exist.[^5g^, ^73^, ^74^, ^75^] However, it seems counterintuitive that these should have no significant effect on the UV/Vis shift. This fact also conclusively explains the following observations in the literature. Solid complexes of **B30** derivatives with HBD solvents such as 1,2‐ethanediol and methanol have been investigated by X‐ray structure analysis.[^73^, ^74^, ^75^] These stoichiometrically well‐defined complex compounds are strongly coloured. Unfortunately, UV/Vis absorption spectra in solid state are not reported for these complexes. The colour of the solid compounds is similar to that of the original dye in the solid state; that is, their colour does not correspond to that of the respective solution. As an example, the solid **B30**/methanol complex is green,^[75]^ but the solution of **B30** in methanol is red.^[5e]^

As a consequence, three independent experimental findings, namely

- correlations of *E*_T_(30) with *D*_HBD_ or *N*, Figure 6 and Figure S19;
- current results of Sander's group;^[9]^ and
- the non‐conformity of the colour of the solid **B30**/alcohol complexes compared to that of the solution,^[75]^ empirically show that the *E*_T_(30) parameter of the HBD solvent is not a function of the hydrogen bridge bonding strength.

Furthermore, the results of quantum‐chemical calculations are sometimes inconsistent or even contradictory, especially to explain the difference of non‐HBD and HBD solvents which show the same dielectric permittivity.[^76^, ^77^, ^78^, ^79^, ^80^] Possible hydrogen bonding complexes of **B30** are theoretically studied by quantum chemical calculations to support the Laurence HBD scale.[^43^, ^80^]

However, the intermolecular hydrogen bonding network structures in the volume of the so‐called HBD‐solvents have a greater influence on overall polarity (global polarity) than dipole‐dipole interactions in non‐HBD solvents. This is the real reason for the special character of HBD solvents. This property is particularly important for water and polyols, as ^129^Xe NMR experiments clearly show.

The special properties of polyhydric alcohols in comparison to monohydric alcohols with regard to *D*_HBD_ recognised in this work are supported by independent literature studies, especially because the number of OH groups per molecule proves to be an important parameter.^[81]^ In addition, the hydrogen bond structure of polyhydric alcohols is very similar to each other. Hydrophobic interactions operate in both cases, but in different ways for polyhydric and monohydric alcohols.[^81^, ^83^] For instance, polyhydric alcohols stabilise and favour the native collagen structure, while monohydric alcohols diminish the degree of order.^[82]^ However, there are still some open questions regarding the quantification of the van der Waals forces of the hydrogen bond network of polyhydric alcohols.

The unique role of water is clearly explained by its high molar concentration, the highest of all common HBD solvents. Only this fact is the reason for the high *E*_T_(30) or the largest lg*k*_1_ value in solvolysis reactions. Any other explanation for water, such as “highest hydrogen bond donating ability” is nonsense and contradicts reality. The structure of water is substantially determined by its three‐dimensional hydrogen‐bond network, which is very dynamic because the hydrogen bonds are constantly being formed and broken.[^84^, ^85^, ^86^, ^87^] Other polar solvents form far smaller clusters, because these solvents are less cohesive compared to water.^[84]^ The average hydrogen bond lifetime of water is about 1 ps and the lifetime of wobbling OH groups is shorter than 200 fs.^[85]^ Therefore, hydrogen bonds are continually broken and reformed. Despite the highly dynamic water structure, the electronic transition of **B30** is faster than the water dynamic. However, the UV/Vis spectrum in solution registered the amount of about 10^20^ molecules of **B30**. Each has a slightly different environment. The entire dye collective thus measures the average of the geometries and the associated polarity of the surrounding hydrogen bond network. Hence, there is no information about the distribution of the geometries in the water or polyhydric alcoholic solvents. The high average binding energy of the hydrogen bonds is compensated by the entropy gain resulting from the bending and breaking of the hydrogen bonds. This process is different for each monohydric and polyhydric alcohol, as dielectric spectroscopy shows.[^87^, ^88^, ^89^, ^90^] Therefore, one can assume that a certain hydrogen bond density distribution is present and differs for monohydric and polyhydric alcohols.

Note that water has a small overall oscillator strength in the UV spectral region, since it does not contain C−H groups. From the viewpoint of electromagnetic coupling as an important or main factor influencing *E*_T_(30), its high *E*_T_(30) value is therefore not surprising. Cooperative hydrogen bonding effects are particularly noticeable with TFE and HFIP as special solvents^[43d]^ which is reflected in their higher physical densities compared to normal alcohols in relation to the molecular weight. Strong hydrogen bonds within the solvent clusters of TFE and HFIP increase the OH density significantly. It is quite conceivable that both effects, that is, the solute‐solvent and solvent‐solvent interactions, represent a collective HBD property for special solvents as suggested for TFE and especially for HFIP.[^46^, ^47^, ^48^, ^49^, ^50^, ^51^]

The molar concentration *N* or *D*_HBD_ is dependent on the density; this in turn is directly related to the refractive index due to the Gladstone‐Dale or Lorentz‐Lorenz relationship.[^10^, ^41^, ^45^] Therefore, it is not scientifically justified to separate the two influencing variables because they are always coupled with each other.

Consequently, both the original classification and determination of the HBD property of alcohols and water as proposed by Kamlet‐Taft and other authors should be abandoned,[^5a^, ^18^, ^19^, ^43^] as the physical meaning of these published parameters does not really relate to acid‐related hydrogen‐bond‐donating properties of the individual solvent molecule. Instead, the solvatochromic parameters reflect a collective measure determined preferentially by the global polarity of the hydrogen bond network and/or by the electromagnetic coupling between solute and solvent and, small in proportion, by hydrogen bond complexation. As long as there is no real HBD parameter available that truly correlates with an acid scale, the use of multi‐parameter equations (see Equations (S3) and (S4), Supporting Information) cannot be scientifically justified.

In following studies,^[53]^ the examination of various polarity scales such as Kosower's *Z*, Drago's *S*, the Gutmann acceptor number *AN*, Catalan's *SA* and the Laurence *α*_1_ scale[^5^, ^18^, ^19^, ^43^, ^54^, ^55^, ^56^] by means of *D*_HBD_ will be presented.

## Experimental Section

1,2,4‐butanetriol in highest purity grade was purchased from Alfa Aesar. Reichardt‘s dye **B30** was provided by Prof. Dr. Ch. Reichardt, University of Marburg. The UV/Vis spectroscopic investigations were carried out using a Cary 60 UV/Vis from Agilent Technologies. **B30** was first dissolved in 0.3 mL1,2,4‐butanetriol. Due to the high viscosity, the **B30** dyes is only sparingly soluble at RT. This solution‘s UV/Vis absorption spectra were recorded in cuvettes made of special optical glass with a light path of 2 mm at 22 °C UV/Vis spectra see Figure S21, Supporting Information part.

## Supporting Information Summary

The Supporting Information section contains 30 pages with numerous literature data on physical properties of alcohols (Table S3, p. 12‐15). The data are arranged in chronological order in line with the results in the main manuscript. The Table of Content is given below:

1. Introduction

1.1 Additional aspects on global polarity from literature (p. 1–2)

1.2. Linear solvation energy relationships (LSER) (p. 2)

2. Result and Discussion

2.1. Aspects and solvent size and molar volume as a function of structure of alcoholic solvents (p. 3–5)

2.2. Data used for correlation analyses (including Tables S1 and S2) (p. 6–7)

2.3. Correlation of *E*_T_(30) as a function of molar concentration of the alcoholic solvents (p. 8)

2.4. Correlation of *f*‐factors with the density of alcoholic solvents (p. 9–11)

2.5. Correlations of Σn as a function of density for alcohol derivatives (including Table S3) (p. 12–20)

2.6. Aspects of the effective *D*_HBD_ parameters for 2,2,2‐trifluoroethanol (TFE) and 1,1,1,3,3,3‐hexafluoro‐isopropanol (HFIP) (including Table S4) (p. 20–21)

2.7. Analysis of δ ^129^Xe NMR data with respect to solvent properties (p. 21–23)

  2.7.1. ^129^Xe NMR Data discussion from literature (p. 23)

2.8. Correlation of *D*_HBD_ with kinetic data (including Tables S5, S6 and S7) (p. 23–28)

2.9. Experimental details of UV/Vis measurements (p. 28)

3. References (p. 28–30)

## Conflict of interest

The authors declare no conflict of interest.

1

## Supporting information

As a service to our authors and readers, this journal provides supporting information supplied by the authors. Such materials are peer reviewed and may be re‐organized for online delivery, but are not copy‐edited or typeset. Technical support issues arising from supporting information (other than missing files) should be addressed to the authors.

Supporting Information

## Data Availability

The data that support the findings of this study are available in the supplementary material of this article.
