# Supplementary material for: The Global Polarity of Alcoholic Solvents and Water – Importance of the Collectively Acting Factors Density, Refractive Index and Hydrogen Bonding Forces
Source: ChemistryOpen. 2022 Oct 25;11(10):e202200140. doi: 10.1002/open.202200140 (PMC9596611; doi:10.1002/open.202200140)
Supplement: Supplementary file 1 — Supporting Information [file OPEN-11-e202200140-s001.pdf]

ChemistryOpen

Supporting Information

The Global Polarity of Alcoholic Solvents and Water – Importance of the Collectively Acting Factors Density, Refractive Index and Hydrogen Bonding Forces

Stefan Spange,* Nadine Weiß, and Thomas G. Mayerhöfer

Supporting Information

1. Introduction part

1.1. Additional aspects on global polarity from literature

If one considers established theories based on electrostatic forces to define global polarity in terms of physical measurands,[1–8] there is an assignment problem. The question arises as to which variable best describes this property. For instance, when using the Lorentz-Lorenz equation (eq. 1) as a basis, it is reasonable to apply the f(*n*) term with respect to V_m to describe a measurand relating to the mean polarizability A_m.[1]

$$f(n) \; = \; = \frac{A_m}{V_m} \tag{S1a}$$

$$f(n) \sim \rho/M = N \tag{S1b}$$

However, for organic solvents whose individual molecules have a static dipole moment, the *Debye* equation (eq. 2) is applicable which shows that P_m (the molar polarization) of solvent molecules increases steadily with V_m or the average dipole moment μ.

$$P_m = \frac{\varepsilon_r - 1}{\varepsilon_r + 2}\frac{M}{\rho} = \frac{N_A}{3\varepsilon_0}(\alpha + \frac{\mu^2}{3kT}) \tag{S2}$$

N_A is the Avogadro constant. The μ term is a vectorial quantity that reflects the average of all dipole moments orientation due to thermal motion.

There are also established theories for describing solvent effects on the UV/Vis-spectra of compounds based on the principles of eqs. 2 and 3.[2–8]

Note that it is often stated that the molar refraction derived from the Lorentz-Lorenz relation is a measure of dispersion forces, which is not correct. This misunderstanding of the Lorentz-Lorenz relation may be a result of the possibility to determine the dipole moment of a molecule from the difference of molar polarization and molar refraction. Based on the Kramers-Kronig relations, however, the index of refraction in a non-absorbing spectral region and, with it, the molar refraction, is a measure of the oscillator strengths of excitations at higher frequency and, therefore, not of dispersion forces.[9]

Considering the Lorentz-Lorenz equation (eq. 2) or the Debey equation (eq. 3) each of the physical quantities (n_D^{20}-refractive index, A_m the molar refractivity, P_m the molar polarization, M - molar mass, α - the electrical polarizability, ρ - physical density, μ - static dipole moment, ε_r - the relative static permittivity) or the molar volume V_m = M/ρ = 1/*N*; with *N* the molar concentration can be correlated with a measurand. However, this way of thinking is a serious pitfall.

Generally, for LSER (Linear Solvation Energy Relationships),[10–13] the most important point to consider is that only measured quantities that are linearly proportional to an energy equivalent may be linearly correlated with each other. Thus, the UV/Vis energy of a solvatochromic dye such as **B30** (see eq. 1) is linearly dependent on *N*, but inversely proportional to V_m. This crucial aspect is not always considered and misleading interpretations can result.[5] Note that from the perspective of electromagnetic coupling as cause of solvatochromism, the linear dependence can only be an approximation, but obviously a very good one.

Due to the complexity of the problem, various LSER-based empirical solvent concepts have emerged which are easy to handle and seem to provide qualitatively reliable statements during the last decades. Reichardt addressed exactly this problem in terms of discrimination between theoretical and empirical concepts.[14] Furthermore, Langhals provided a beneficial evaluation for advantageous application the Lorentz-Lorenz function f(*n*) (see eq. 2a) compared to the Kirkwood function f(ε) = (ε_r - 1)/(ε_r +2) (see eq. 3) in comparison to empirical concepts for organic solvents.[12c]

The most appropriate and widely used empirical multiparameter concepts come from Kamlet-Taft (KAT) and Catalán, which describe the solvent property using three and four parameters, respectively.[10,11]However, one must bear in mind that these polarity terms were all artificially invented and have taken on a life of their own. The real problem is that the actual physical meaning of the empirically derived specific polarity scales is not always consistent with their definition.[14]
The challenge is that there are concrete relationships between empirical polarity data and material properties (density, refractive index) that are ignored for organic solvents, because they seem to hold only to individual solvent families.[3, 12,13] For ionic liquids, such relationships have recently been clearly demonstrated and accepted, although they apply to specific anion classes.[4]
However, it is not the problem of the definition of the HBD term, but of the correct interpretation of the determined parameters such as the KAT α value that seem to refer to this property. A related problem arises for the Catalán *SA* (solvent acidity) scale.
Regardless of the many statements and interpretations, if a linear dependence of any empirically constructed polarity parameter with the molar concentration *N* ($N = \rho/M$) exists, a systematic change of a volume-determined property such as the static dielectric constant ε_r and/or the refractive index (*n*) depending on structural features should be the reason for this result (eq. 1 and 2).[1–8c] Therefore, a linear correlation of a solvent-dependent chemical property relating to an energy equivalent (lg k_1, $\Delta_R G$, $1/\lambda_{max}$) as a function of *N* within a homologous or reaction series clearly indicates that nonspecific effects or at least collective properties play the dominant role. Note that from the perspective of electromagnetic coupling, the energy equivalent just refers to the exciting radiation, but not directly to the energy difference of two states of matter, because both are no longer equivalent. The difference is the larger the stronger the involved oscillators are. Additionally, optical influences like reflection and interference can lead to spectral shifts.[9]
With this respect, the decisive problem is that it is often difficult to distinguish whether an R-O-H bond acts as a dipole or by hydrogen bond formation, because the effect on the physical property of the interacting partner in terms of a spectroscopic measure can be the same.[16,17] For both cases, the interaction as in the example of an OH-group with a carbonyl group would lead to the same changes in the IR as well as ^{13}C NMR spectrum.

1.2. Linear solvation energy relationships (LSER)

The Kamlet-Taft (KAT) LSER-approach considers the three independent empirical polarity parameters: α, the hydrogen bond donating (HBD) property, β, the hydrogen bond accepting (HBA) property and π^* the dipolarity/polarizability property.[10] (*XYZ*) reflects the result of a solvent dependent chemical process and $(XYZ)_0$ is the reference process in a nonpolar medium. Altogether, *a*, *b*, *s*, are solvent independent correlation coefficients. δ is the correction term for the polarizability, that is 0.5 for halogen-containing and 1.0 for aromatic solvents. In the most cases the simplified LSER eq. S3 is used for multiple square correlation analyses.[10]

$$(XYZ) = (XYZ)_0 + a\alpha + b\beta + s(\pi^* + d\delta) \qquad \text{(S3)}$$

Later, the LSER concept of Catalán (eq. S4) was predicted as a complementary alternative to the KAT solvation parameters from eq. S3.[11]

$$P = (P)_0 + aSA + bSB + sSP + eSdP \qquad \text{(S4)}$$

P is the result of a solvent dependent chemical process and $(P)_0$ is the reference process in the gas phase. *a*, *b*, *s*, and *e* are solvent independent correlation coefficients according to eq. S3. The determinations of the individual polarity parameters *SA* (the solvent acidity), *SB* (the solvent basicity), *SP* (solvent polarizability), and *SdP* (solvent dipolarity) are based on UV/Vis measurements of selected solvatochromic reference compound.
Altogether, the empirical polarity parameters of eq. S3 and eq. S4 were determined with dissolved solvatochromic probe molecules. Their concentration being approximately 10^{-3} mol/L.[1–3]
The parameters presented in eqs. 3 and 4 can be qualitatively divided into two categories. While π^*, *SdP*, and *SP* are parameters determined by the solvent volume (nonspecific effects), α, β, *SA* and *SB* should reflect molecular properties (specific effects) of the individual solvent molecule.
This means that π^*, *SdP*, and *SP* are clearly physically defined in terms of established theories regarding eq. 3 and 4.
However, linear correlations of α, β, *SA* and *SB* with the molar concentration *N*, recognized for special solvent families, indicate dipolar portion on the parameter (see introduction main text). 4, [3,4]

2. Results and Discussion part

2.1. Aspects and solvent size and molar volume as a function of structure of alcoholic solvents

For reasons of comprehensibility, the scientific arguments for using eq. 2 of the main text as the basis are briefly justified. The molar concentration *N* indicates how many molecules are present on average in a defined volume. It is assumed that the solvent molecules are statistically equally distributed in the liquid. If we consider the number of monohydric alcohol molecules in a defined volume, then this value also corresponds to the average number of OH-groups in this volume. The situation becomes different if we consider 1,2-ethanediol. Then two OH-groups are to be considered per molecule. Furthermore, the distribution of OH-groups in the volume of a dihydric alcohol is then no longer uniform, but two at a time in the very small volume of the single solvent molecule at infinite dilution. This inhomogeneity of the OH-group distribution increases with the number of OH-groups in a molecule. Therefore, the OH-group density of an alcoholic solvent in relation to *N* depends on both the number of OH-groups per molecule and the overall number of molecules. Intermolecular interactions between the alcohol molecules are still not considered in this basic consideration. But they play a crucial role, because the actual distance between the solvent molecules is strongly determined by the van der Waals radius (r_w) ($2r_w$ – hard sphere diameter) and the attracting hydrogen bonds forces between the molecules.[18,19] That is vividly demonstrated by the dependence of the molar volume on the sum of the individual solvent molecules in eq. S5, modified for this study, originally taken from the ref.[18]

$$1/N = V_m = M/\rho = V_m \cdot N_A = 4/3\pi \cdot N_A \cdot (r_w + \Delta)^3 \quad \text{(S5)}$$

The Δ is the separation term on the length scale. The Δ term is due to the repulsion between the electrons, it forces the molecules apart. Δ becomes smaller the stronger the interaction between the molecules by non-repulsive forces. The smaller Δ, the larger the density of the solvent regardless of the molar mass.

The contribution of Δ to the actual global interaction strength between the solvent molecules can be seen in the concrete difference between the experimentally measured molar solvent volume and the theoretically calculated molar volume, *i.e.* the sum of the van der Waals volumes of the individual molecules.

$$V_m = N_A \cdot 4/3\pi \cdot r_w^3 \quad \text{(S6)}$$

The real scenario for pure solvents also becomes particularly clear when V_m is plotted as cubic function against the hard sphere diameter for water, the C_1 to C_{11} primary alcohol homologous series, 1,2-ethanediol and glycerol.

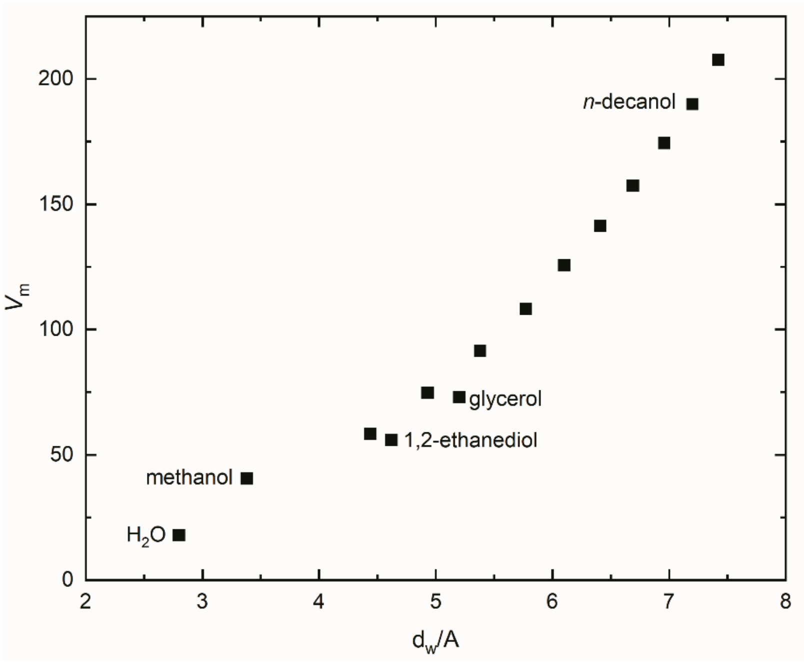

Figure S1. Plotting of V_m as cubic function of the hard sphere diameter $2r_w = d_w$ (in A = 100 pm) of the individual solvent molecule for primary C_1–C_{11} alcohols, water, 1,2-ethanediol and glycerol according to eq. 6. Data are taken from the ref.[18-21]

The stronger the interaction between the molecules, the smaller the distance and the greater the noticeable shrinkage, which leads to an additional increase in density. The relative volume shrinkage increases with increasing number of OH-groups: glycerol (18 %) > 1,2-ethanediol (15 %) > methanol (11 %) measured in water.[18]

Thus, the V_m for water, 1,2-ethanediol and glycerol is lower than expected from the theory of additivity according to eq. 6. Thus, Δ for polyhydric alcohols is lower than for monohydric ones (eq. 5). If one plots V_m as a function of d_w for a greater set of solvents including voluminous molecules as hexadecane (see Fig. S2) a cubic function is also obtained as expected from the theory (eq. 6). The crucial aspects regarding the apparent shrinkage of V_m for polyhydric alcohols are also recognizable in this diagram. It is also notable that molecules with lower density (*n*-pentane) are located above the line of the theoretical function (see Fig. S2).

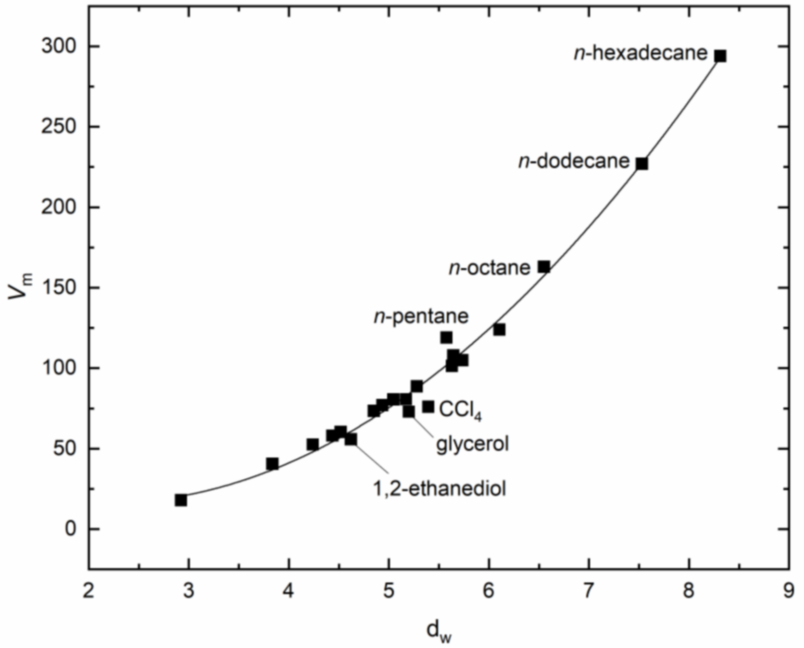

Figure S2. Plotting of V_m as a function of the hard sphere diameter of the individual solvent molecule for several types of organic solvents. Data from refs.[18-21]

$V_m = 7.285\ d_w^2 - 31.237\ d_w + 49.501$ (S7)
$n = 22$, $r = 0.994$, $sd = 7.132$

Solvents with higher density (CCl_4) show lower V_m values, and solvents with low density (*n*-pentane) show larger V_m as theoretically expected as discussed in main text.
As the conclusion of this special chapter shows, the density has a double influence, since both *N* is determined by it by definition and the distance between the molecules is shaped by it. This double influence is not coupled, but takes place independently of each other. In the main text of this study we will show that density exerts a manifold influence on the volume-determined measurand of a solvent system. It is assumed that special solutes are surrounded by an infinite number of solvent molecules. Therefore, aspects of the surface tension of alcohols are not yet considered in this basic approach.

2.2. Data used for correlation analyses

Table S1 $E_T(30)_{measured}$ – $E_T(30)$ value for solvents are taken from: C. Reichardt, T. Welton, in *Solvents and Solvent Effects in Organic Chemistry*, 4th Edition, Wiley-VCH, Weinheim, 2010, pp. 455ff and $E_T(30)_{theoretically\ calculated}$ by eq. 7b from main text – $E_T(30)_{calculated} = 383.19\ N + 45.66$, f – weighting factor with respect to primary alcohols and ρ – density of alcohol derivatives.

alcohol	$E_T(30)_{measured}$	$E_T(30)_{calculated}$	f	ρ [g/cm^3] (20°C)
methanol	55.1	55.10	1.000	0.790
ethanol	51.9	52.23	0.994	0.789
1,2-ethanediol	56.3	52.50	1.070	1.110
2-methoxyethanol	52.0	50.53	1.030	0.965
2-ethoxyethanol	51.0	49.61	1.028	0.930
2-butoxyethanol	50.0	48.57	1.030	0.900
2-ethanolamine	51.8	52.06	0.995	1.010
2,2,2-trifluoroethanol	59.8	50.98	1.173	1.360
2,2,2-trichloroethanol	54.1	49.60	1.170	1.550
2-chloroethanol	55.1	51.37	1.070	1.200
2-propanol	48.4	50.63	0.956	0.786
allylalcohol	51.9	51.23	1.013	0.854
(±)-1,2-propanediol	54.1	50.90	1.063	1.040
1,3-propanediol	54.9	50.98	1.076	1.060
1-methoxy-2-propanol	48.6	49.57	0.980	0.920
glycerol	57.0	50.89	1.120	1.260
1,1,1,3,3,3-hexafluoroisopropanol	62.1	49.30	1.257	1.600
	65.3	49.30	1.313	1.600
1-butanol	49.7	49.80	0.998	0.810
2-butanol	47.1	49.80	0.945	0.806
2-methyl-2-propanol	43.3	49.75	0.870	0.790
2-methyl-1-propanol	48.6	49.27	0.986	0.802
1-amino-2-propanol	50.1	50.60	0.990	0.960
1-pentanol	49.1	49.18	0.998	0.814
2-pentanol	46.5	49.18	0.945	0.812
3-pentanol	45.7	49.20	0.930	0.815
2-methyl-1-butanol	48.0	49.20	0.980	0.815
3-methyl-1-butanol (*i*-amyl)	49.0	49.18	0.990	0.810
2-methyl-2-butanol (*tert* amyl)	41.0	49.20	0.830	0.815
3-methyl-2-butanol, (*sec* isoamyl)	45.7	49.20	0.930	0.818
1-hexanol	48.8	48.75	1.001	0.814
1-methoxy-2-propanol	48.6	49.57	0.980	0.920
2-amino-1-butanol	50.2	49.71	1.010	0.943
3-ethyl-3-pentanol	38.5	48.40	0.790	0.820
3-ethyl-2,4-dimethyl- 3-pentanol	37.9	47.92	0.790	0.800
2,4-dimethyl-3-pentanol (DMP)	40.1	48.46	0.827	0.829
cyclopentanol	47.0	49.76	0.940	0.950
cyclohexanol	47.2	49.32	0.960	0.962

1,4-butanediol	53.5	49.99	1.070	1.020
1,2-butanediol	52.6	49.95	1.050	1.000
1,3-butanediol	53.5	49.95	1.060	1.010
2,3-butanediol	51.8	49.95	1.030	0.987
1,2,4-butanetriol	55.2*	49.95	1.105	1.185
benzyl alcohol	50.4	49.34	1.020	1.040
2-phenylethanol	49.5	48.86	1.010	1.020
3-phenylpropanol	48.5	48.47	1.000	1.040

*measured for this work

Table S2 Alcoholic solvents, water and D_2O considered in this study. $E_T(30)$ (ref.[S17]), N and D_{HBD} values calculated from $D_{HBD} = N(f_p n_p + f_s n_s + f_t n_t) = N\Sigma n$ with the f factors of Tab. S1.

solvent	$E_T(30)$ [kcal/mol]	N [mol/cm^3]	D_{HBD} [mol/cm^3]	$f(n_D^{20})$
water	63.1	0.05541	0.05541	0.2057
D_2O	62.8	0.05526	0.05526	
methanol	55.4	0.02466	0.02466	0.20345
ethanol	51.9	0.01715	0.01715	0.22015
2-methoxyethanol	52.0	0.0127	0.013	
2-ethoxyethanol	51.0	0.01032	0.01032	
2-(*n*-butoxy)ethanol	50.0	0.0076	0.0076	
allyl alcohol	51.9	0.01463	0.01463	0.24883
1-propanol	50.7	0.01331	0.01331	0.23381
2-propanol	48.4	0.01298	0.0125	0.2312
2-methyl-1-propanol (Isobutanol)	48.6	0.01079	0.0107	
1-butanol	49.7	0.01093	0.01093	0.23272
2-butanol	47.1	0.01093	0.01038	0.24124
2-methyl-2-propanol	43.3	0.01066	0.00927	0.23543
2-methyl-1-propanol	49.0	0.0092	0.0092	0.24001
1-pentanol	49.1	0.00919	0.00919	0.24723
2-pentanol	46.5	0.00919	0.00868	0.24526
3-pentanol	45.7	0.00925	0.0089	0.247
1-hexanol	48.8	0.00803	0.00803	0.25201
1-heptanol	48.5	0.00706	0.00706	0.25517
1-octanol	48.1	0.00637	0.00637	0.2578
1-nonanol	47.8	0.00575	0.00575	0.2599
1-decanol	47.7	0.00524	0.00524	0.26199
1-undecanol	47.6	0.00482	0.00482	0.26355
1-dodecanol	47.5	0.00445	0.00445	0.265
cyclopentanol	47.0	0.01103	0.0104	
cyclohexanol	47.2	0.0096	0.0092	
triethanolamine	53.7	0.00757	0.0227	
diethanolamine	53.1	0.0104	0.0208	
ethanolamine	51.8	0.0167	0.0167	
1-amino-2-propanol	50.1	0.0129	0.0085	
2-amino-1-butanol	50.2	0.01057	0.01057	
2-amino-4-methyl-1-pentanol	49.3	0.0077	0.0077	
benzyl alcohol	50.4	0.00962	0.00962	
1-phenylethanol	46.7	0.00827	0.00545	
2-phenylethanol	49.5	0.00835	0.00835	
3-phenylpropanol	48.5	0.00734	0.00734	
2-methyl-1-butanol	48.0			
3-methyl-1-butanol (isoamylalcohol)	49.0	0.00919	0.00919	0.2456
2-methyl-2-butanol (*tert*-amylalcohol)	41.0	0.00923	0.00766	0.245
3-ethyl-3-pentanol	38.5	0.00705	0.00557	0.258
3-ethyl-2,4-dimethyl- 3-pentanol	37.9	0.0059	0.00466	0.264
2,4-dimethyl-3-pentanol (DMP)	40.1	0.00731	0.00604	0.2557

solvent	$E_T(30)$ [kcal/mol]	N [mol/cm³]	D_{HBD} [mol/cm³]	$f(n_D^{20})$
diethyleneglycol monomethylether	–	0.0848	0.0848	
1,2-ethanediol	56.3	0.01785	0.0357	0.25927
1,2-propanediol	54.1	0.01367	0.027	0.25958
1,3-propanediol	54.9	0.0139	0.0278	0.2627
1,2-butanediol	52.6	0.01121	0.0219	0.2624
1,3-butanediol	52.8	0.01121	0.02219	0.26355
1,4-butanediol	53.5	0.01132	0.0226	0.26667
1,2,4-butanetriol	55.2	0.0112	0.033	0.2805
1,5-pentanediol	51.9	0.0095	0.019	0.2687
1,6-hexanediol	–	0.00812	0.0162	
1,8-octanediol	–	0.0062	0.0124	
2,3-butanediol	51.8	0.01121	0.0219	0.26178
glycerol	57.0	0.01368	0.0405	0.28134
1,2,6-hexanetriol	–	0.00827	0.0244	
1-methoxyglycerol	53.7	0.01055	0.0175	
1,3-dimethoxyglycerol	50.5	0.00839	0.0055	
diethylene glycol	53.8	0.0113	0.0226	
triethylene glycol	52.8	0.00732	0.0146	
tetraethylene glycol	52.2	0.00576	0.0115	
2-methylpentane-2,4-diol	–	0.00496	0.0089	
3,5,5-trimethylhexane-1-ol	–	0.00568	0.00556	
**Alcohols with electron withdrawing groups**				
2-cyanoethanol	59.6	0.0149	[b]	
2-chloroethanol	55.1	0.0149	[b]	
2,2,2-trichloroethanol	54.1	0.01037	[b]	
2-mercaptoethanol	53.6	0.0143	[b]	
propargylalcohol	55.7	0.017	[b]	
lactic acid ethylester	51.1	0.00872	[b]	
tartaric acid diethylester	50.8	0.0058	[b]	
2,2,2-trifluoroethanol (TFE)	59.8	0.01389	0.0419 [a]	
1,1,1,3,3,3-hexafluoro-2-propanol (HFIP)	62.1<br>65.3	0.0095	0.038[a]<br>0.057[a]	
2,2,3,3-tetrafluoro-1-propanol (TFP)	59.4	0.01113	[c]	

[a] calculated by $D_{HBD} = N\Sigma n$, according to $\Sigma n = 4.486\,\rho - 2.785$ (eq. 1 in the main text). TFE with $\rho = 1.29$ ($\Sigma n = 3$) and HFIP with $\rho = 1.6$ ($\Sigma n = 6$).
[b] unreliable, because of the electron withdrawing group, see main text.
[c] not determined.

2.3. Correlation of $E_T(30)$ as a function of molar concentration of the alcoholic solvents

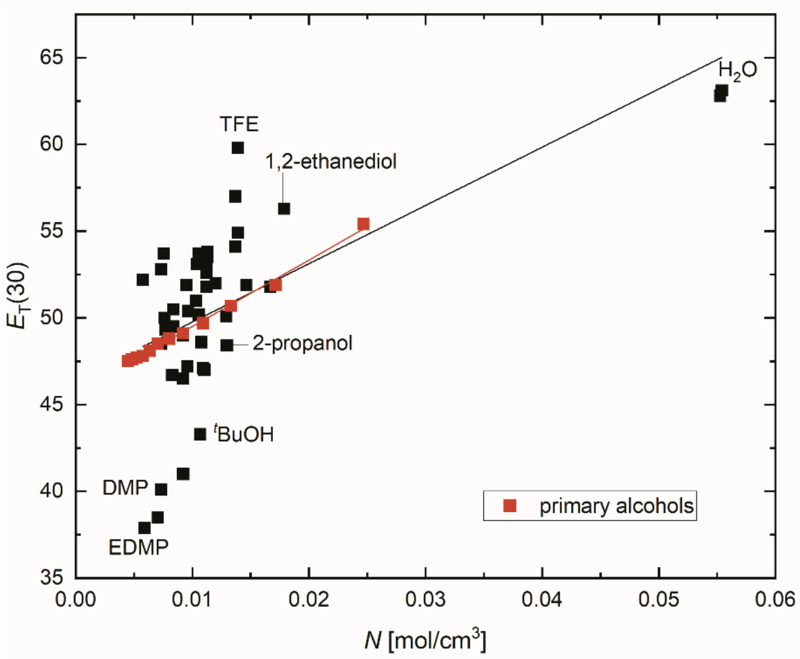

Figure S3a Plotting $E_T(30)$ as a function of the molar concentration N for alcoholic HBD solvents from literature. The 12 red points refer to monohydric primary alcohols from C_1 to C_{12}. Altogether, the black points belong to the other alcohols. The five points of sterically hindered alcohols located between 2-methyl-2-propanol (^{t}BuOH) and 3-ethyl-2,4-dimethyl-3-pentanol (EDMP) belong to 3-methyl-2-butanol, 2-methyl-2-butanol, 3-ethyl-3-pentanol and 2,4-dimethyl-3-pentanol (DMP).

$$E_T(30) = 335.897\ N + 46.401 \qquad (S8)$$

$n = 43$, $r = 0.608$, $sd = 4.268$

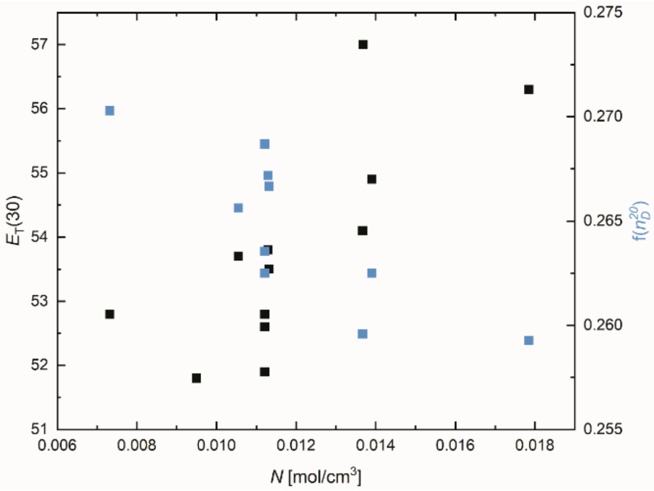

Figure S3b. Correlation of $E_T(30)$ and $f(n_D^{20})$ (blue) as a function of N for dihydric and polyhydric alcohols of the C_2-, C_3- and C_4-families.

2.4. Correlation of *f*-factors with the density of alcoholic solvents

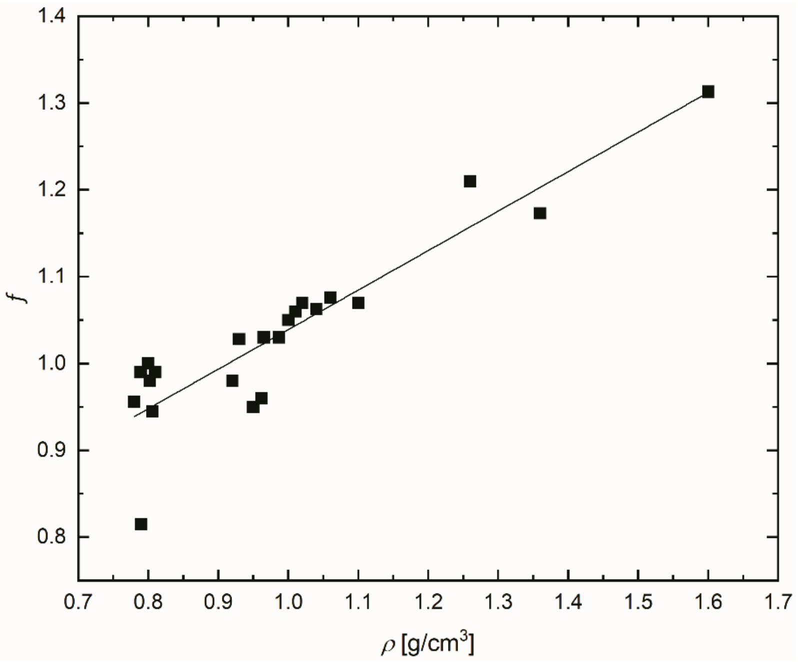

Figure S4 Correlation of *f* of the alcohol as a function of its density ρ including C_2, C_3 and C_4 alcohols.

$f = 0.451\ \rho + 0.587$ (S9)
$n = 23$, $r = 0.904$, $sd = 0.044$
$n = 18$ (without *t*-butanol and cyclic alcohols), $r = 0.971$

Ethanol derivatives
The situation is striking for 2-alkoxyethanol derivatives and 2,2,2-trifluoroethanol. Then the measured $E_T(30)$ are larger than expected according to eq. S10. The reason for this particular result is clearly due to the density of the alcohol derivative. There is a significant linear correlation between *f* and the density of the ethanol derivatives, with TFE well included.

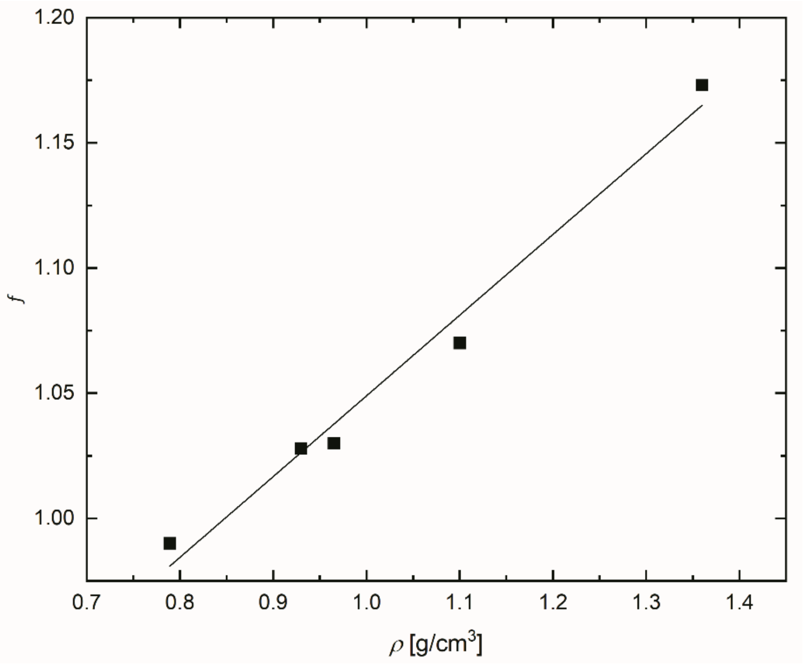

Figure S5a Correlation of *f* as a function of density for C_2-alcohols including glycol and TFE.
$f = 0.225\ \rho + 0.727$ (S10)

n = 5 (C_2-alcohol derivatives), r = 0.989, sd = 0.011

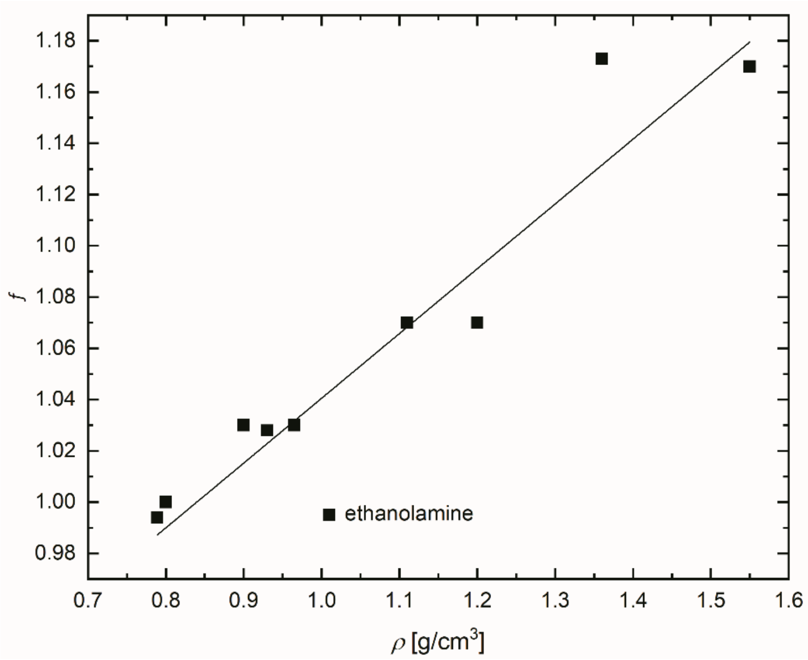

Figure S5b Correlation of f as a function of density for ten C_2-alcohols including chloroethanol derivatives.

$f = 0.253\ \rho + 0.788$ (S11)

n = 10 (C_2-alcohol derivatives from Fig. S3), r =0.927, sd = 0.025

Propanol derivatives

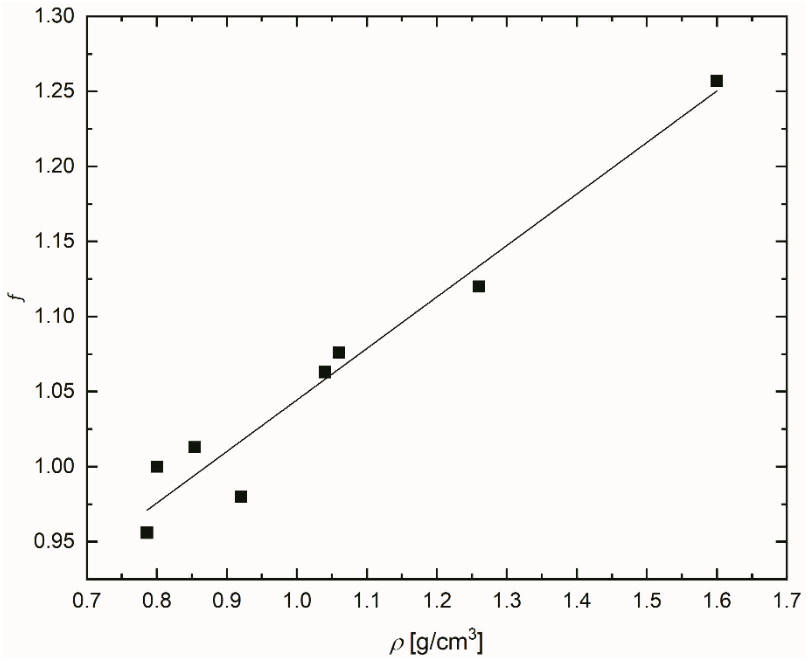

Figure S6 Correlation of f as a function of density for C_3-alcohols including 1-propanol, 2-propanol, allyl alcohol, 1,2-propane-diol, 1,3-propanediol, 1-methoxy-2-propanol glycerol, and HFIP (underlaid with E_T (30) = 62.1).[7e]

$f = 0.343\ \rho + 0.701$ (S12)

n = 8 (C_3-alcohol derivatives), r = 0.974, sd = 0.022

Butanol derivatives

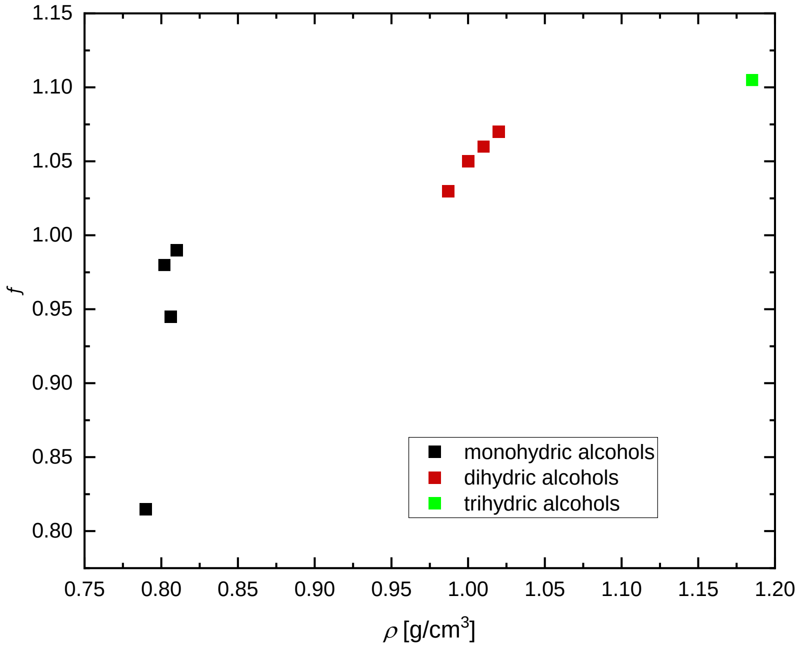

Figure S7 Correlation of *f* as a function of density for C_4-alcohols including monohydric (black squares) and dihydric (red squares) alcohol derivatives as well as 1,2,4-butanetriol (green). Each solvent group shows a separate line.

2.5. Correlations of Σn as a function of density for alcohols derivatives

Basically, the density of monohydric alcohol isomers decreases constantly from primary > secondary > tertiary alcohol for propanol- and butanol-isomers, but not for monohydric pentanol- and hexanol-isomers (see Tab. S3). Therefore, the molar volume $V_m = 1/N$, which relates to the overall polarizability according to the Lorenz-Lorentz equation, increases from the primary to the tertiary alcohol only for propanol- and butanol-derivatives. For monohydric pentanol isomers, the density does not alter significantly as a function of molecular structure, but the 3-methyl-2-butanol has the largest density of the monohydric pentanol isomers. For the monohydric hexanol isomers the situation is also unclear and difficult to quantify (see Tab. S3). Then, among the 17 various monohydric hexanol isomers, the 3-methyl-2-pentanol shows the largest density in spite of the tertiary OH-group. This is a clear indication that the attractive intermolecular van der Waals energy contributions of the alkyl groups are close to the effect of the intermolecular hydrogen bonds.

Furthermore, the search for physical densities of polyhydric hexanol- and pentanol-derivatives in the literature is tedious. Data see Tab. S3. There are hardly comprehensive references available which report on properties of those type of compounds. There are only a few physical data available without literature references from different providers.

The degree of the actual folding of the alkyl substituent is also a factor that has an influence on the intermolecular van der Waals interaction. This scenario becomes increasingly important for branched alcohols containing longer alkyl chains. The reason for this peculiarity is the influence of the ovality (O) of the alkyl chain. O is the ratio of the real surface area to the minimum surface area of the conformationally flexible alkyl chain. Thus, the approach in this work is only valid for short alkyl groups, since the spherical volume of the group is approximately equal to the real molecular volume, but O increases with increasing length of the alkyl chain. Therefore, the interpretation of polarity measurements of pentanol, hexanol or longer alkyl chain alcohol-derivatives compared to propanol and butanol isomers must be handled with care. All these points play a role in the interpretation of the polarity values of various alcohols.

Table S3 List of physical data of various alcohol families including monohydric and polyhydric derivatives. Physical densities and refractive indices at 20 °C. Free space in table indicates that reliable data are not available.

number C atoms/ OH-groups	Alcohol derivative	M [g/mol]	ρ [g/cm³]	*N* [mol/cm³]	n_D^{20}
	water	18.00	0.998	0.0554	1.333
1	methanol	32.04	0.79	0.02466	1.329
**C_2 family**					
2	ethanol	46.07	0.789	0.01715	1.359
3	2-methoxyethanol	76.09	0.965	0.01268	1.4024
6	2-butoxyethanol	118.17	0.9	0.00762	1.419
2/2	1,2-ethanediol	62.07	1.11	0.01788	1.4318
8	2-hexoxyethanol	146.23	0.89	0.00608	1.4291
8	2-phenoxyethanol	138.16	1.1	0.00796	1.534
2	2,2,2-trifluoroethanol	100.04	1.39	0.01389	1.3
**C_3-family**					
3	1-propanol	60.09	0.8	0.01331	1.384
3	2-propanol	60.09	0.786	0.01298	1.377
3	allyl alcohol	58.08	0.854	0.01474	1.4135
3/2	1,2-propanediol	76.09	1.04	0.01367	1.4324
3/2	1,3-propanediol	76.09	1.06	0.0139	1.4383
3/3	1,2,3-propanetriol (glycerol)	92.09	1.26	0.01368	1.4746
3	cyclopropanol	58.08	0.917	0.015788	1.4129
3	1,1,1,3,3,3-hexa-fluoro-2-propanol (HFIP)	168.05	1.6	0.0095	1.275
**C_4-family**					
4	1-butanol	74.12	0.81	0.01093	1.3988
4	2-butanol	74.12	0.806	0.01087	1.3978
4	2-methyl-1-propanol	74.12	0.802	0.0108	1.3955
4	2-methyl-2-propanol	74.12	0.781	0.0105	1.387
4/2	1,4-butanediol	90.12	1.02	0.01132	1.446
4/2	1,3-butanediol	90.12	1.01	0.01121	1.44

4/2	1,2-butanediol	90.12	1.01	0.01121	1.438
4/2	2,3-butanediol	90.12	0.987	0.01095	1.431
4/3	1,2,4-butanetriol	106.12	1.185	0.0112	1.473
4/3	1,2,3-butanetriol	106.12	1.18	0.01112	1.4670
4/4	1,2,3,4-butanetetrol (erythritol)	122.12	1.45	0.01187	1.536
**cyclic derivatives**					
4/1	1-methylcyclopropanol	72.11	1.265	0.0175	1.414
4/1	cyclobutanol	72.11	0.921	0.01277	1.435
**C_5 family**					
5/1	1-pentanol	88.15	0.814	0.00923	1.41
5/1	2-pentanol	88.15	0.812	0.00921	1.405
5/1	3-pentanol	88.15	0.815	0.00924	1.4104
5/1	2-methyl-2-butanol	88.15	0.815	0.00924	1.4052
5/1	3-methyl-2-butanol	88.15	0.818	0.00928	1.409
5/1	2-methyl-1-butanol	88.15	0.82	0.0093	1.4107
5/1	2,2-dimethylpropan-1-ol (neopentyl-alcohol)	88.15	0.812	0.00921	1.3915
5/2	1,5-pentanediol	104.15	0.99	0.0095	1.4499
5/2	1,2-pentanediol	104.15	0.97	0.00931	1.439
5/2	1,3-pentanediol	104.15	0.986	0.009467	1.4472
5/2	1,4-pentanediol	104.14	0.986	0.009467	1.447
5/2	2,2-Dimethyl-propane-1,3-diol	104.15	0.978	0.00939	1.443
5/3	1,2,5-pentanetriol	120.15	1.14	0.0095	1.484
5/3	1,1,1-Tris(hydroxy-methyl)ethane	120.15	1.159	0.00965	1.486
5/4	1,2,3, 4-pentanetetrol (1-deoxy-D-ribitol)	136.15	1.331	0.009775	1.523
5/4	1,1,5,5-pentanetetrol	136.15	1.336	0.0098	1.526
5/4	2,2,4,4-pentanetetrol	136.15	1.31	0.00962	1.531
5/4	2,2-bis(hydroxy-methyl)-1,3-propanediol (pentaerythrite)	136.15	1.396	0.01025	1.548
5/5	xylitol	152.15	1.52	0.00999	1.571
**cyclic C_5 derivatives**					
5/1	cyclopentanol	86.13	0.949	0.01102	1.4521
5/2	1-methylcyclobutanol	86.13	0.997	0.01157	1.482
5/2	1,3-cyclopentanediol	102.13	1.094	0.01071	1.483
5/2	*cis*-1,2- cyclopentanediol	102.13	1.235	0.01209	1.478
5/2	*trans*-cyclopentanediol	102.13	1.235	0.01209	1.547
**C_6 family, acyclic derivatives**					
6/1	1-hexanol	102.18	0.82	0.008027	1.418
6/1	2-hexanol	102.18	0.810	0.00793	1.414
	3-hexanol	102.18	0.819	0.008015	1.416
	2-methyl-1-pentanol	102.18	0.826	0.008084	1.419
	3-methyl-1-pentanol	102.18	0.824	0.00806	1.418
	4-methyl-1-pentanol	102.18	0.813	0.007956	1.414
	2-methyl-2-pentanol	102.18	0.81	0.00793	1.411
	3-methyl-2-pentanol	102.18	0.831	0.008133	1.421
	4-methyl-2-pentanol	102.18	0.808	0.0079	1.415
	2-methyl-3-pentanol	102.18	0.824	0.008064	1.418
	3-methyl-3-pentanol	102.18	0.829	0.008113	1.418
	2,2-dimethyl-1-butanol	102.18	0.828	0.0081	1.4188
	2,3-dimethyl-1-butanol	102.18	0.81	0.00793	1.415
	2,3-dimethyl-2-butanol	102.18	0.823	0.00805	1.417
	3,3-dimethyl-1-butanol	102.18	0.815	0.007976	1.414
	3,3-dimethyl-2-butanol	102.18	0.812	0.00795	1.415
6/1	2-ethyl-1-butanol	102.18	0.833	0.00815	1.4224
6/2	3-methyl-1,5-pentanediol	118.18	0.974	0.00824	1.454
6/2	1,6-hexanediol	118.18	0.96	0.00812	1.457
6/2	2,5-hexanediol	118.18	0.96	0.00812	1.447
6/2	1,2-hexanediol	118.18	0.951	0.00805	1.442
6/3	1,2,6-hexanetriol	134.17	1.11	0.00827	1.478

6/5	1,2,4,6-hexanetetrol	150.17	1.30	0.00866	1.519
6/6	(2*R*,3*S*,4*R*,5*S*)-hexane-1,2,3,4,5,6-hexol (galactitol)	182.17	1.47	0.00807	1.553
6/6	sorbit (70% aqueous solution)	-	1.29		1.4581/1.449
	sorbitol	182.17	1.596	0.00876	1.597
6/6	sorbitol (solid hydrate)	200.17	1.49	0.0074	(1.57)*
6/6	mannitol (solid)	182.17	1.52	0.008344	1.597
**cyclic C_6 family including substituted cyclopentanols**					
6	1-methylcyclopentanol	100.16	0.904	0.00903	1.443
6	3-*trans*-methyl-cyclopentanol	100.16	0.947	0.00945	1.446
6	cyclohexanol	100.16	0.962	0.0096	1.4641
6/2	*cis*-1,2-cyclohexanediol	116.16	1.156	0.00995	1.4644
6/2	*trans*-1,2-cyclohexanediol	116.16	1.157	0.00996	1.526
6/2	*trans*-1,4-cyclohexanediol	116.16	1.156	0.009952	not reported
6/2	1,3-cyclohexanediol (*cis-trans* mixture)	116.16	1.156	0.009952	1.526
6/4	(1*R*,2*R*,4*S*,5*S*)-1,2,4,5-cyclohexanetetrol	148.16	1.6	0.0108	1.64
6/3	1,3,5-cyclohexanetriol (isomer mixture)	132.16	1.356	0.01026	1.58
6/6	inositol (isomer mixture)	180.16	1.4–1.65	–	1.4544-1.546[a]
6/6	myo-inositol	180.16	1.752	0.00972	
6/6	epi-inositol (1*R*,2*R*,3*R*,4*S*,5*S*,6*S*)-cyclohexane-1,2,3,4,5,6-hexol)	180.16	2.038	0.0113	1.784
**C_7 family**					
7	1-heptanol	116.88	0.819	0.0070	1.422
7	2-heptanol	116.88	0.817	0.00699	1.414
7	3-heptanol	116.88	0.818	0.00699	1.42
7	4-heptanol	116.88	0.82	0.00702	1.421
7	2,4-dimethyl-3-pentanol	116.88	0.829	0.00709	1.425
7/2	1,7-heptanediol	132.20	0.951	0.0072	1.455
7/2	2,3-heptanediol	132.20	0.9	0.0068	1.448
7/3	1,2,3-heptanetriol	148.20	1.1	0.0074	1.48
7/4	1,1,7,7-heptanetetrol	164.20	1.2	0.00731	1.513
7/5	5-*O*-methyl-myo-inositol (sequoyitol)	194.18	1.56	0.00803	1.588
(6C+O)/4	3-(2,3-dihydroxypropoxy)-propane-1,2-diol (diglycerol)	166.17	1.28	0.0077	1.489
7/2	1-(2-Hydroxyethyl)-cyclopentanol	130.18	1.092	0.00839	1.4815
**C_8 family**					
8	1-octanol	130.23	0.83	0.00637	1.4295
8	2-octanol	130.23	0.82	0.00629	1.426
8	3-octanol	130.23	0.82	0.00629	1.426
8	4-octanol	130.23	0.818	0.00628	1.426
8	3-ethyl-3-pentanol	130.23	0.824	0.00633	1.43
8	2,4-dimethylpentan-3-ol	130.23	0.829		1.425
8/2	1,8-octanediol	146.23	0.939	0.00642	1.454
8/2	1,2-octanediol	146.23	0.937	0.00641	1.452
8/2	1,3-octanediol	146.23	0.937	0.00641	1.4554
8/2	2,2,4-trimethylpentan-1,3-diol	146.23	0.937	0.00641	1.4513
8/2	2,5-dimethyl-2,5-hexanediol	146.23	0.939	0.00642	1.453
8/3	1,2,8-octanetriol	162.23	1.057	0.00652	1.481
8/3	1,4,7-octanetriol	162.23	1.054	0.0065	1.479
**nonanol family**					

9	1-nonanol	144.25	0.83	0.00575	1.431
9	2-nonanol	144.25	0.82	0.00568	1.429
9	3-nonanol	144.25	0.824	0.00571	1.4289
9	4-nonanol	144.25	0.82	0.00568	1.43
9	3-ethyl-2,4-dimethyl- 3-pentanol	144.25	0.854	0.00592	1.442
9	3,5,5-trimethyl-1-hexanol	144.25	0.82	0.00592	1.432
9	diisobutylcarbinol-2,6-dimethyl-4-heptanol	144.25	0.809	0.00575	1.423
9/2	1,9-nonanediol	160.25	0.95	0.00593	1.456
9/2	1,2-nonanediol	160.25	0.929	0.005797	1.454
9/3	1,2,3-nonanetriol	176.25	1.036	0.005878	1.48
**decanol family**					
10	1-decanol	158.28	0.83	0.00524	1.4372
10	2-decanol	158.28	0.827	0.005225	1.434
10/2	1,10-decanediol	174.30	0.891 (1.08)	0.0062 (0.00533)	1.457
10/2	1,2-decanediol	174.30	0.922	0.0053	1.456
10/3	1,2,10-decanetriol	190.28	1.02	0.00536	1.479
	1H,1H,10H,10H-perfluoro-1,10-decanediol	462.123	1.673	0.00362	1.313
**higher alcohols**					
12	*n*-dodecanol	186.34	0.831	0.00446	1.443
14	*n*-tetradecanol	214.39	0.84	0.00392	1.4454
15 (Fp. 41)	*n*-pentadecanol	228.42	0.842	0.00368	1.446
16	cetylalcohol	242.44	0.842	0.00347	1.46
18	stearylalcohol	270.49	0.83	0.00307	1.46
(15C+4O)/7 O indicates ether bond rinstead C bond	(3-[3-[3-[3-(2,3-dihydroxypropoxy)-2-hydroxypropoxy]-2-hydroxypropoxy]-2-hydroxypropoxy]propane-1,2-diol (pentaglycerol)	388.41	(1.252) 1.358	0.00349	1.535
20	*n*-eicosanol	298.5	0.8 (4°C)		1.435
24	n-tetracosanol	354.7	0.839	0.0033	1.456
28	n-octacosanol	410.76	0.841 (20°C)	0.00205	1.485
$(2/1)_n$	polyvinylalcohol	-	1.19–1.31		1.477–1.483

[a] *N* is extrapolated from aqueous solutions to 100 % solid
[b] there are nine stereoisomers to consider; see text. Not considered in correlations.
[c] From CAS date base, https://www.chembk.com/en/chem/Inositol.
[d] https://www.chemicalbook.com/ChemicalProductProperty_DE_CB3457869.htm.

Several comments to data of Tab. S3:

Finding the appropriate physical density data for certain diols and polyols was sometimes quite complicated. Often, the relative density is given, but not mentioned in detail like in WIKIPEDIA.
Furthermore, there are nine stereoisomers for inositol. The reported data for inositol differ mainly in density due to isomer mixtures are present in solid form, but only slightly or hardly in refractive index. Therefore, it made sense to use data from only one source. In addition, these compounds are all hygroscopic, which is probably the reason for the variance of the density data.
If the refractive index data of *e.g.* aqueous sorbitol solutions are extrapolated to 100% content solid, the value obtained is somewhat (little) higher than the value measured for the pure solid. Therefore, the actual measured density or refractive index of the solid is contaminated by traces of water from the air. Since sorbitol is strongly hygroscopic compared to mannitol, which is not hygroscopic at all, the data from different sources in the literature for sorbitol differ considerably. Thus, no reliable data for sorbitol are really available.

Another example, two different density values 1.252 and 1.358 are reported for pentaglycerol. Correlations with *n^D20* within alcohol series do not allow an exact assignment according to the Gladstone-Dale relationship (J.H. Gladstone, T.P. Dale, T. P. Researches on the refraction, dispersion and sensitiveness of liquids. Phil. Trans. Royal Soc. London 1864, 153, 317–343). It is likely that 1.358 is the correct one due to water bond to the solid can affect the density.

http://www.chemnet.com/cas/de/50-70-4/Sorbitol.html
NPCS Board of Consultants & Engineers: Industrial Alcohol Technology Handbook. ASIA PACIFIC BUSINESS PRESS Inc., 2010, ISBN 81-7833-143-8, pp. 268.
http://www.chemspider.com/Chemical-Structure.99339.html
http://ursula.chem.yale.edu/~chem220/chem220js/STUDYAIDS/isomers/RS14272/inositol.html
3-[3-[3-[3-(2,3-dihydroxypropoxy)-2-hydroxypropoxy]-2-hydroxypropoxy]-2-hydroxypropoxy]propane-1,2-diol
https://www.sciencedirect.com/topics/chemistry/cis-inositol
https://www.guidechem.com/encyclopedia/6-deoxy-l-galactitol-dic296970.html#id_-975635171.
https://www.chemsrc.com/en/cas/74742-09-9_92310.html

Correlations of Σn as function of density for several alcohol families

Ethanol and propanol derivatives

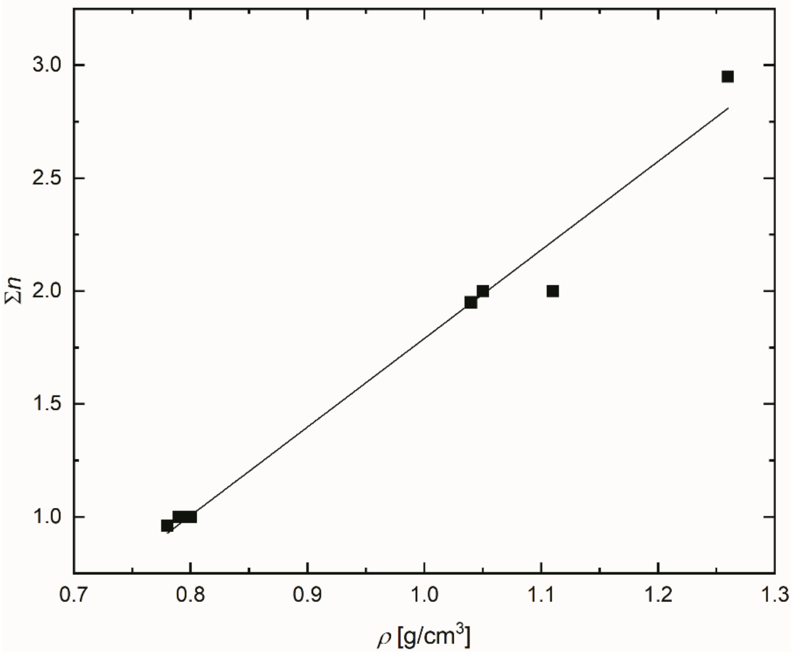

Figure S8a Correlation of Σn as a function of the density (25 °C) for the five propanol derivatives (2-propanol, 1-propanol, 1,2-dihydroxypropanol, 1,3-dihydroxypropanol, glycerol) including 1,2-ethandiol and ethanol.

$\Sigma n = 3.921\ \rho - 2.131$ (S13a)
$n = 7$, $r = 0.987$, $sd = 0.119$
TFE: $\rho = 1.29 \rightarrow N = 2.9$
HFIP: $\rho = 1.6 \rightarrow N = 4.14$

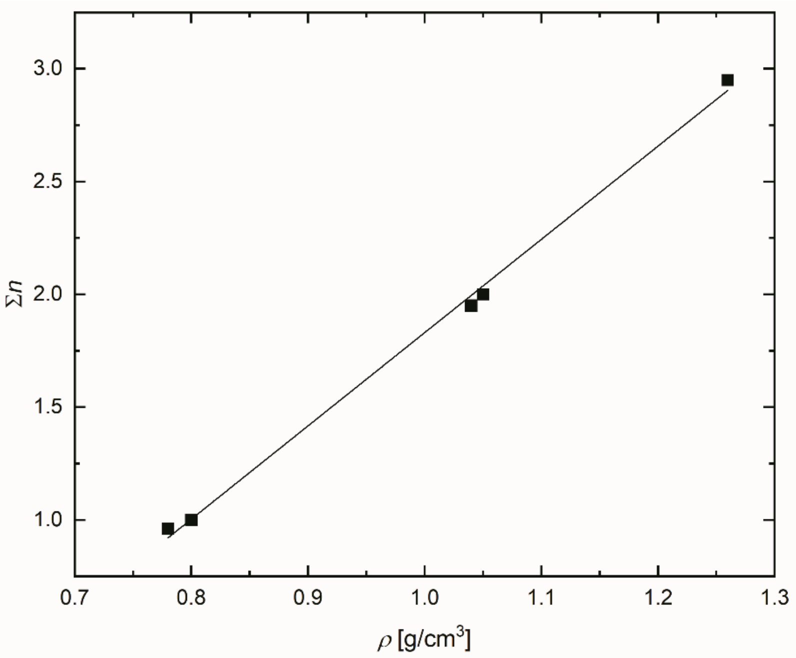

Figure S8b Correlation of Σn as a function of the density (25 °C) for the five propanol derivatives (2-propanol, 1-propanol, 1,2-dihydroxypropanol, 1,3-dihydroxypropanol, glycerol).

$\Sigma n = 4.134\ \rho - 2.304$ (S13b)

n = 5, r = 0.998, sd = 0.048

Pentanol derivatives

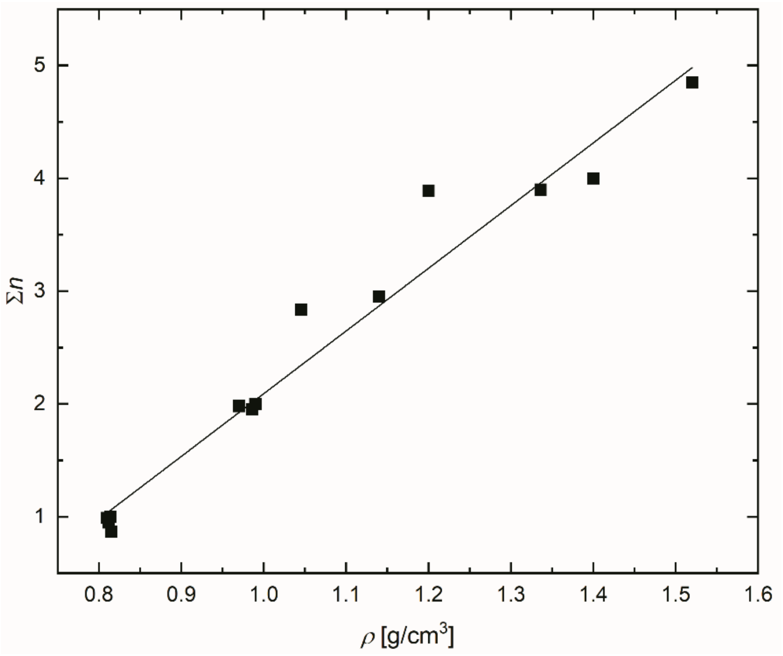

Figure S9 Correlation of Σn as a function of the density (20 °C) of 14 derivatives of the C_5-alcohol family including 1,1,5,5-pentanetetrol and pentaerythrite. 2,3,4-pentanetriol and 1,2,4,5-pentanetetrol are the two deviants above the line, but included in the correlation equation.

$$\Sigma n = 5.558\,\rho - 3.468 \qquad (S14)$$

n = 15, r =0.983, sd = 0.271

Hexanol derivatives

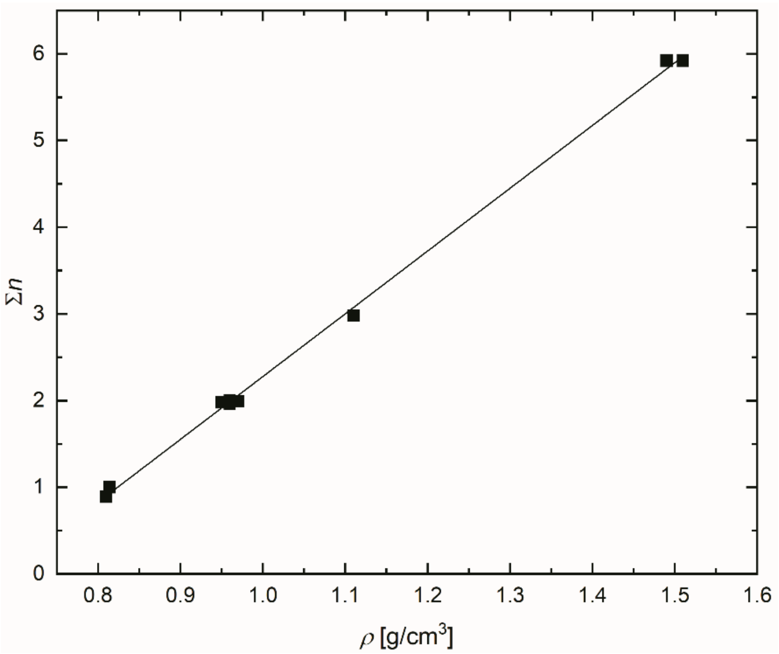

Figure S10 Correlation of Σn as a function of density of nine derivatives of the C_6-alcohol family including mannitol and sorbitol.

$$\Sigma n = 7.241\,\rho - 4.965 \qquad (S15)$$

n = 9, r =0.999, sd = 0.071

Heptanol derivatives

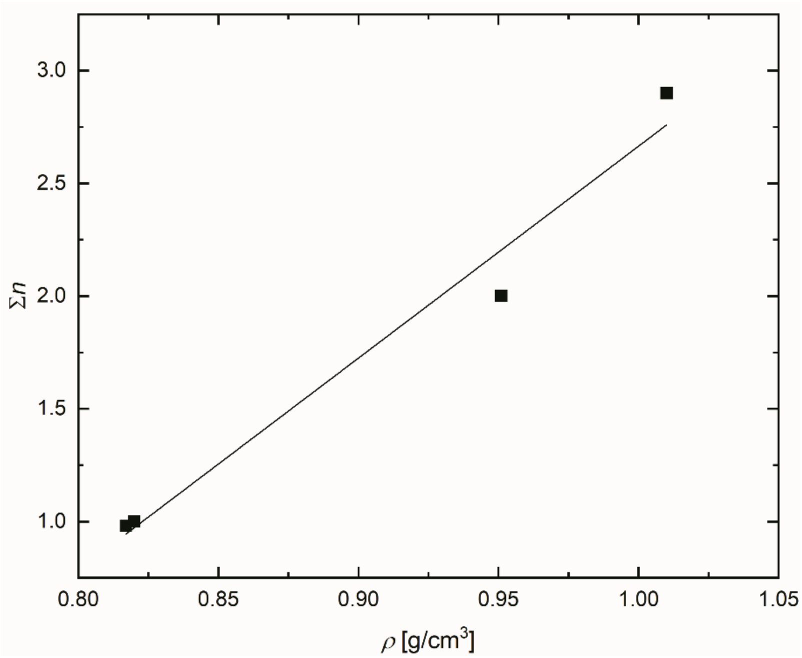

Figure S11 Correlation of Σn as a function of density for four derivatives of the heptanol family.

$\Sigma n = 9.400\ \rho - 6.735$ (S16)
n = 4, r = 0.981, sd = 0.178

The significant linear increase of Σn with ρ is a clear prove of the importance of the number of OH-groups per volume for the physical alcohol volume property.
It is remarkable how accurate the linearity of Σn as a function of density is for polyhydric butanol- and pentanol derivatives including various constitutional isomers. From Figs. S13-S15 can be concluded that the greater the number of carbon atoms of the alcohol, the more the Σn-term is influenced by the density. This trend is clearly confirmed when mono- and polyhydric hexanol and heptanol derivatives are included. The correlation coefficients for the hexanol and heptanol families are preferentially determined by the densities of the polyhydric alcohols rather by the monohydric alcohol isomers, because for monohydric isomers is no differentiation recognizable due to the mismatch of Σn and density as discussed before.
Therefore, the use of the individual position factor from eq. 13 (main text) is only unrestrictedly suitable for propanol and butanol derivatives.

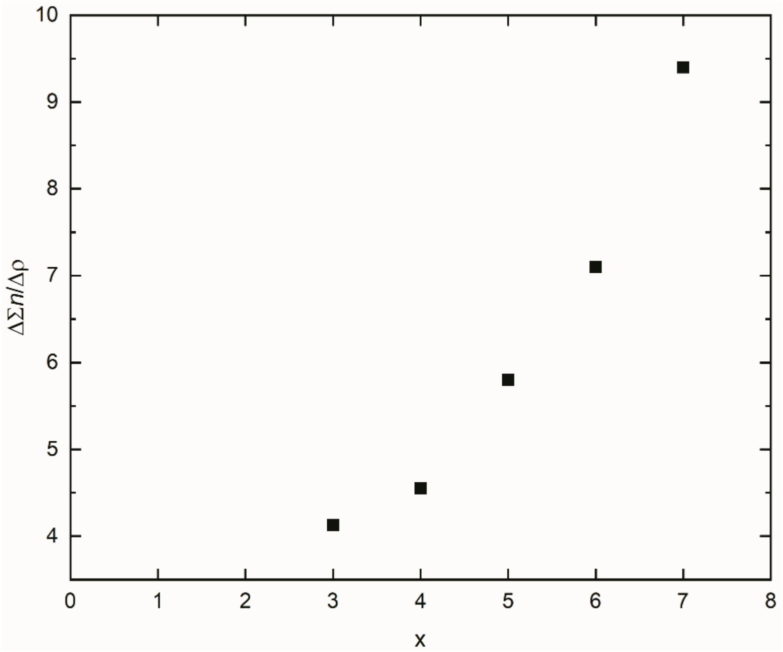

Figure S12 Dependence of the sensitivity $\Delta\Sigma n/\Delta\rho$ (slope coefficient) on the number of carbon atoms X of the alcohol family. The $\Delta\Sigma n/\Delta\rho$ value for heptanol derivatives is only based on four (*r* = 0.985) date pairs.

The established relationships of Σn as a function of density are of great importance, because they only consider the number and position of the OH-group regardless the molar mass of the molecule. The increase of the slope coefficient $\Delta\Sigma n/\Delta\rho$ with number of carbon atoms x is a strong indication for the dominant influence of the oscillating strength of the alcohol derivative.

2.6. Aspects of the effective D_{HBD} parameters for 2,2,2-trifluoroethanol (TFE) and 1,1,1,3,3,3-hexafluoroisopropanol (HFIP)

In the main text has been suggested that the actual OH-group density of TFE (ρ = 1.39g/mL) and HFIP (ρ = 1.596 g/mL) is significantly higher than reflected by the *N* value.
Thus, the microscopic structure of the OH-groups and their number at the molecular level per volume is crucial. Assuming that the approach eq. 13 (main text) applies to molecules of similar size, apparent Σn parameters for TFE, HFIP and TFP (2,2,3,3-tetrafluoro-1-propanol) can be theoretically calculated, because the van der Waals radii of hydrogen and fluorine of organic molecules do not vary so much. Hence, apparent Σn values were calculated from the correlation of Σn as a function of ρ. Results see Tab. S4.
However, several reported $E_T(30)$ values for HFIP are secondary $E_T(30)$ values calculated by correlation equations.[22-24] The UV/Vis spectrum of a fluorine-substituted **B30** dye can also be measured in HFIP. Such secondary $E_T(30)$ values should not be used for correlation analyses regarding physical properties of HFIP. Fortunately, related $E_T(30)$ values for HFIP can be directly measured by the dichloro derivative **33** of **B30**.
Dye **33** is the so called „Wolfbeis-dye“ (see Scheme 1, main text) that contains two chloro-substituents in place of the two phenyl rings at the negatively charged phenolate ring.

Dye **33** can be used in stronger acidic environments due to its lower basicity (pK_a = 4.76). These measured polarity data are designated as $E_T(33)$ in correspondence to the literature.[22-24] The correlation of $E_T(33)$ as a function of D_{HBD} for primary alcohols looks very good (see eq. S17).

$$E_T(33) = 278.32\ D_{HBD} + 55.755 \quad \text{(S17)}$$
n = 6 (for primary alcohols and water excluding TFE), *r* = 0.975

We have checked $E_T(33)$ as a function of D_{HBD} considering a larger set of alcoholic solvents. UV/Vis data of dye **B33** were taken from literature.

$$E_T(33) = 302.5\ D_{HBD} + 54.89 \quad \text{(S18)}$$

n = 13 (excluding tertiary alcohols), r = 0.944)

Furthermore, there are different correlation equations reported according to $E_T(30) = a\, E_T(30) + b$ in literature.[24]
Already these results show that differences in the coefficient a are often deceptive, because of the different data set used. A deviation of a within a range of 15 % is not significant enough for a physical statement. Thus, there is no real difference in the sensitivity of **B30** compared to dye **33** with respect to the D_{HBD} quantity or N.
Hence, actual D_{HBD} values have been hypothetically calculated for TFE and HFIP by eq. S18. HFIP has been treated as a primary alcohol because too few $E_T(33)$ data of secondary alcohols are reported. Possibly, the calculated Σn value is a little bit too large. These Σn results are compiled in the Table S4.

Table S4 Theoretically calculated apparent Σn solvent parameters for TFE, HFIP and TFP from data of this work and derived from physical results in literature (ref. 46-51, main text).

Parameter	Solvent			Method
	TFE	HFIP	TFP	
Σn	3.29	4.11	3.64	Density correlation
$E_T(33)_{measured}$	69.2	71.5	-	UV/Vis data-
Σn = D_{HBD}/N	3.48	5.95	-	correlation
Σn	3	-	-	X-ray diffraction
Σn		4-6	-	^{1}H NMR, X-ray
Σn		4	-	kinetics
Σn / D_{HBD}	3 / 0.0417	6 / 0.057		UV/Vis data

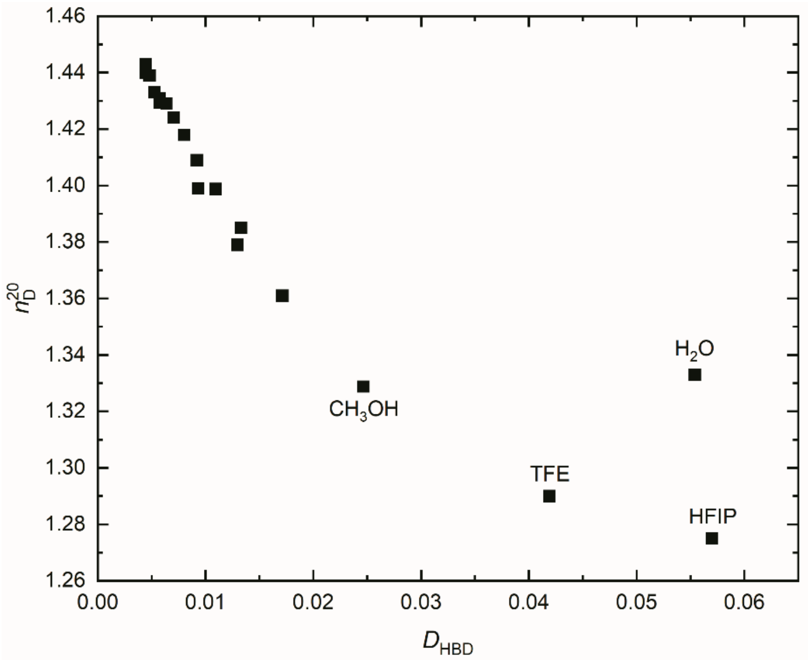

Figure S13 Plotting of the refractive index n_D^{20} as a function of D_{HBD} for primary alcohols and water, including TFE and HFIP with the newly predicted D_{HBD} values based on density consideration.

2.7. Analysis of δ ^{129}Xe NMR data with respect to solvent properties

Correlations of δ ^{129}Xe with the Lorentz-Lorenz function f(n) (eq. S1a) are established indicating the dominance of the disperse part as shown in the pioneering work by Miller [25] (note our criticism of the linkage between the Lorentz-Lorenz function under eq. S1. Chapter 1a, above). The authors themselves have remarked that there fit includes a non-zero constant which it should not have if the Lorentz-Lorenz function could be interpreted as they do.[25]
However, a correlation of δ ^{129}Xe with the Catalán *SP* (solvent polarizability) is recognizable with moderately good correlation quality (see eq. S19).

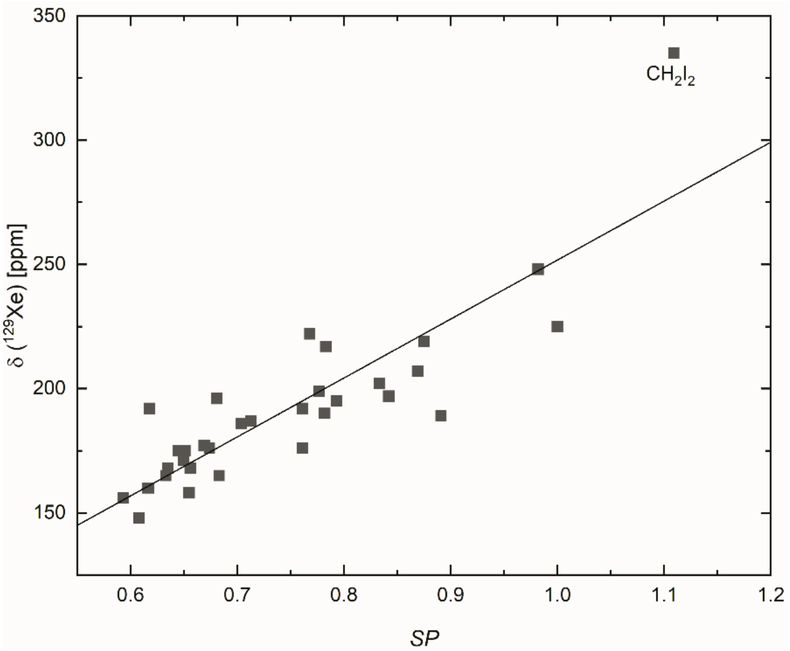

Figure S14 Correlation of δ ^{129}Xe NMR as a function of *SP* (*SP* of CH_2I_2 was determined by us using eq. f(*n*) = 0.359 *SP* + 0.000168, from ref.[11])

$$\delta\ (^{129}\text{Xe})\ [\text{ppm}] = 237.295\ SP + 14.420 \quad \text{(S19)}$$
$n = 33$, $r = 0.865$, $sd = 17.339$

The best qualitative correlation of δ ^{129}Xe, however, is found in the function versus physical density ρ, which is in agreement with literature and theoretically expected according to the Lorentz-Lorenz eq. S1. [25] Note that there is an alternative explanation, based on elementary electromagnetics, since the density of the field lines at a certain frequency is depending on the value of the dielectric function of the solvent, and, thereby, the NMR shift). The excellent classification of diiodomethane within this correlation is particularly noteworthy (see Fig. S15).

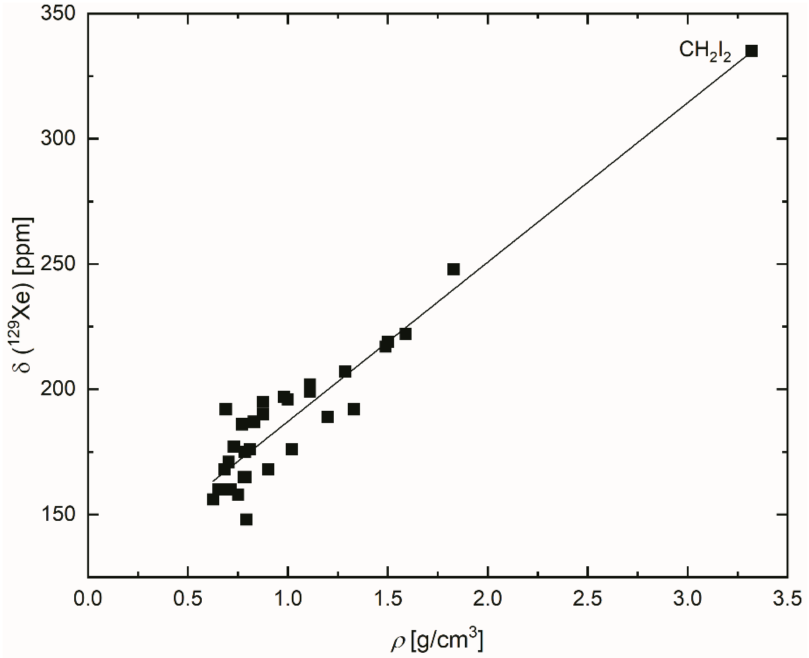

Figure S15 Correlation of δ ^{129}Xe NMR, measured in 32 various solvents,[25] as a function of their densities.

δ (^{129}Xe) [ppm] = 63.621 ρ + 123.484 (S20)
n = 32, r = 0.949, sd = 10.861

2.7.1 ^{129}Xe NMR Data discussion from literature

The interaction enthalpy $\Delta_R H$ of water with Xe is much larger than that of alcohols as shown in the excellent work by Graziano[21] that also explains the role of the high molar concentration and density on physical properties of primary alcohols compared to alkanes. Thus, the three-dimensional hydrogen bonding network in the polyhydric alcoholic solvent or water determines the ability to solvate a non-polar molecule, and not the overall number of atoms per molecule that make up the individual solvent molecule. Therefore, even with non-polar solutes, the inner solvent cavity surface within water has an unprecedentedly strong dispersion influence on the electronic properties. This cannot be explained at all by hydrogen bonds to the substrate, because Xe is unable for hydrogen bond formations with water molecules, but by the totality of van der Waals forces of the global hydrogen bond network is operating as nonspecific share of the global polarity. This interpretation is completely in agreement with ^{129}Xe NMR studies of aqueous urea solutions.[26] With the increasing urea concentration, a slight downfield shift of the ^{129}Xe NMR signal can be observed, indicating a disturbance of the water envelope. In agreement, the $E_T(30)$ value also decreases with increasing urea concentration as expected, [27] indicating that the number of water molecules per volume is reduced. Consequently, polyols and water show an evidently stronger dispersion interaction with a solute compared to small sized monohydric alcohols.

2.8. Correlation of D_{HBD} with kinetic data

Table S5 Rate constants (lg k_1) for the solvolysis reaction of ^{t}BuCl measured in various solvents from different literature sources. D_{HBD}, and Y parameters of the solvents. [28-33]

**Solvent**	D_{HBD}	**lg k_1 [28-32] (^{t}BuCl)**			**Y [27]**
		Abraham/Dvorko	Catalán/Laurence	Dvorko	
water	0.05541	-1.54	-1.55	-1.55	3.493
methanol	0.02466	-6.1	-6.07	-6.07	-1.09
ethanol	0.01713	-7.07	-7.06	-7.06	-2.033
1-propanol	0.01331	-7.13	-7.33	-7.33	
2-propanol	0.0085	-7.83	-7.83		-2.73
2-methyl-propan-1-ol	0.01079	-7.4	-8.3	-8.3	
1-butanol	0.01093	-7.52	-7.28	-7.28	
1-hexanol	0.00803	-7.45			
1-octanol	0.00637	-7.52			
glycerol	0.0364	-4.1	-4.1	-4.1	
1,4-butanediol	0.0226	-5.99	-5.99	-	
2,3-butanediol	0.0147	-6.21	-6.21	-	
1,2-butanediol	0.0186	-6.14	-6.14		
benzyl alcohol	0.00962	?			
1,3-propanediol	0.0278	-5.29			
1,2-propanediol	0.0227	-5.51	-5.51	-	
1,3-butanediol	0.0186	–	-6.05		
1,2-ethanediol	0.03576	-4.6	-4.6		
1,5-pentanediol	0.019	-6.32			
diethylene glycol	0.0226	-5.69			
triethylene glycol	0.0146	-6.0			
cyclohexanol [a]	0.00633	–	-8.07	-8.07	
3-methyl-1-butanol	0.0092	-7.6	–	-	
2-methoxy-ethanol	0.0127	-6.63	–	-	
TFE	0.0419	-3.98	-2.59	-3.98	1.045
HFIP	uncertain for **^{t}BuCl**	-2.7	–	-2.59	
acetic acid	0.0179	-6.7	-6.7	-6.7	-1.675
2-methyl-2-butanol (*tert.*-amyl)	0.00766	-8.77	-8.77	-8.77	
tert. butanol	0.00927	-8.27	-8.39	-8.39	-3.26

aniline	0.011	-6.1	-6.15	-6.15	
formamide	0.0251	-4.4	-4.33	-4.33	0.604
N-methyl-formamide (NMF	0.0169	-6.54		-6.54	
phenol	0.0114	-4.66		-4.66	
N-methylacetamide (NMA)	0.0128	-7.33		-7.33	
HCOOH	0.0265	-2.96		-2.96	2.054

[a] attend, the lg k_1 data for cyclohexanol are confused with them for cyclohexane in Tab. 2 of ref.29 (entry 22 and 44)

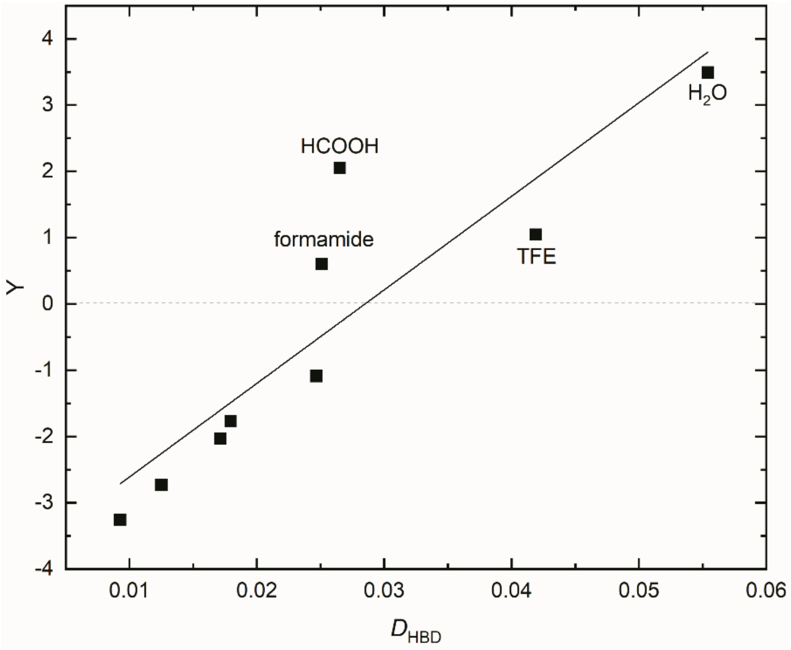

Figure S16 Correlation of Winstein's Y as a function of D_{HBD} for pure alcoholic HBD-solvents (TFE, methanol, ethanol, 2-propanol, and 2-methyl-propane-2-ol) including water, HCOOH, acetic acid, and formamide.

$$Y = 140.124\ D_{HBD} - 4.485 \quad \text{(S21a)}$$

n = 7 (water, alcohols and acetic acid), r = 0.996, sd = 0.208

Especially the very good integration of 2-methyl-propane-2-ol (*tert*-butanol) is highlighting. If HCOOH and formamide are included, the correlation is worsened:

$$Y = 141.142\ D_{HBD} - 4.022 \quad \text{(S21b)}$$

n = 9 (including HCOOH and formamide), r = 0.879, sd = 1.103

For the correlation of same Y with $E_T(30)$ compared to eq. 21a, a clearly worse correlation is found (see eq. S22).

$$Y = 0.339\ E_T(30) - 18.84 \quad \text{(S22)}$$

n = 7 (including formamide), r = 0.943

But it should be noted that HCOOH and acetic acid cannot considered for this correlation because there are no directly measured $E_T(30)$ values available. [14]

Table S6 Polarity data, physical constants and lg k_1 (ref. [29, 34, 35]) for polyhydric alcohol with different carbon/oxygen ratio.

Solvent [(C+O)/OH]-ratio	$E_T(30)$	lg k_1	D_{HBD}	n_D^{20}	ρ [g/cm^3]
1,2-ethanediol [1]	56.3	-4.61	0.0357	1.432	1.15
1,2-propanediol [1.5]	54.1	-5.51	0.027	1.432	1.04
1,3-propanediol [2]	54.9	-5.29	0.0278	1.438	1.05
1,2-butanediol [2]	52.6	-6.14	0.0219	1.38	1.00
1,3-butanediol [2]	52.8	-6.05	0.02219	1.44	1.01
1,4-butanediol [2]	53.5	-5.99	0.0226	1.446	1.02
1,5-pentandiol [2,5]	51.9	-6.32	0.019	1.4499	0.99
2,3-butanediol [2]	51.8	-6.21	0.0219	1.431	1.01
glycerol [1]	57.0	-4.1	0.0405	1.475	1.26
diethylene glycol [2,5]	53.8	-5.69	0.0226	1.447	1.12
triethylene glycol [4,5]	52.8	-6.07	0.0146	1.453	1.10
water	63.1	-1.54	0.05541	1.333	0.99

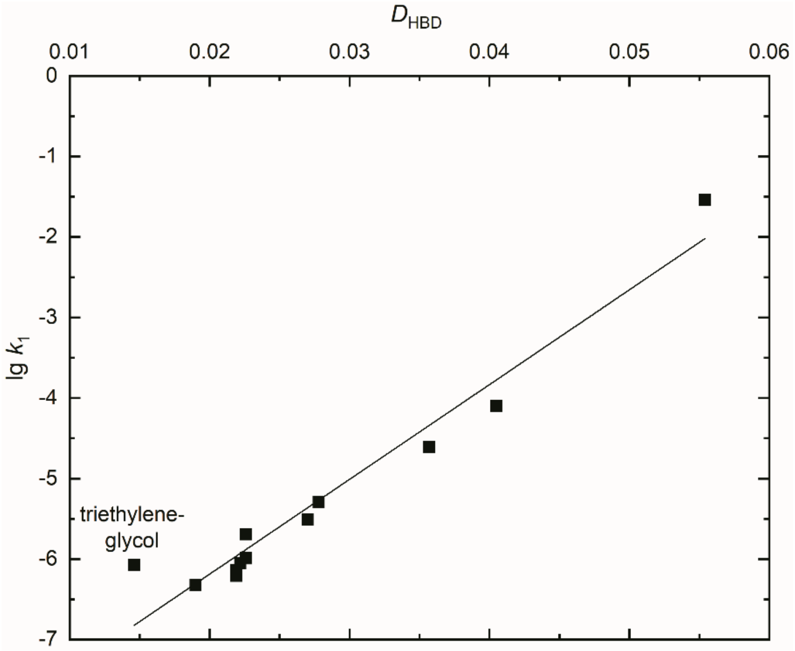

Figure S17 Correlation of lg k_1 as a function of D_{HBD} for glycerol, dihydric alcohols and water.

$$\lg k_1 = 117.713\, D_{HBD} - 8.542$$
$$n = 12,\ r = 0.967,\ sd = 0.340 \qquad (S23)$$

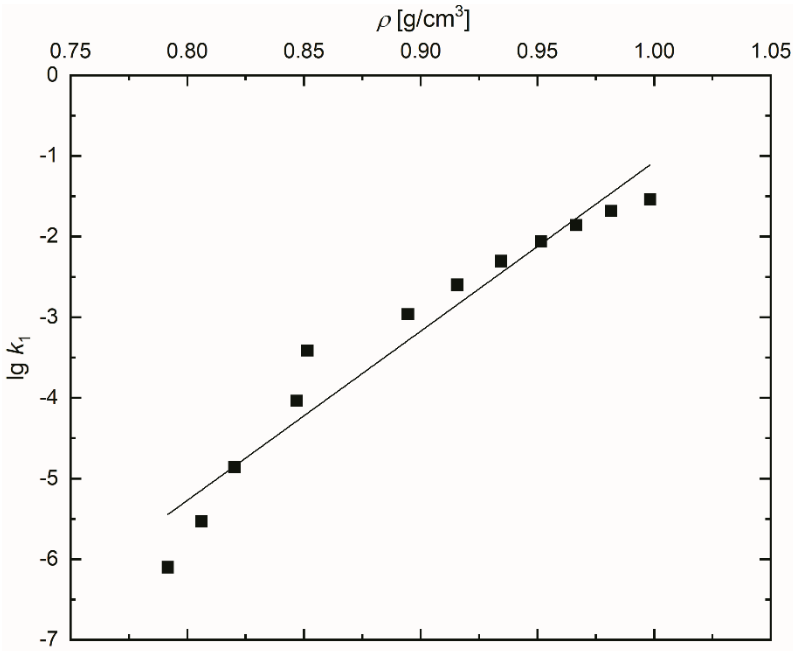

Figure S18 Correlation of lg k_1 as a function of density of the methanol/water mixture (density data from ref.[33, 36])

$$\lg k_1 = 20.998\,\rho - 22.064 \qquad (S24)$$

n = 12 r = 0.965, sd = 0.409

Tab. S7 Kinetic data lgk_1 , E_T(30) and physical constants of the ethanol /water mixtures (ref. 33, 36, 37,38)

Solvent	M (average) g/mol	Density g/cm^3	E_T(30) kcal/mol	N_{AV} a) mol/cm^3	lgk$_1$
0 Ethanol 100% Water	18,015	0,99823	63,1	0,0554	-1,54
7,5	20,12	0,98553	61,9	0,04898	
5	19,414	0,98938	62,4	0,0509	
10	20,8	0,98187	61,68	0,0472	-1,72
15	22,2	0,97514	60,96	0,0437	
20	23,6	0,96864	59,94	0,041	-1,98
30	26,43	0,95382	57,99	0,0361	
60	34,84	0,89113	55,98	0,0256	-3,91
70	37,65	0,86766	54,25	0,023	-4,44
80	40,46	0,84344	53,74	0,02085	-5,03
90	43,28	0,81797	52,95	0,0189	-5,78
94	44,95	0,8138	(52) (ref. 38)	0,018	-6,72
95	44,66	0,8214	52,56	0,0184	-6,32
97,5	45,33	0,79698	52,17	0,01758	
100% ethanol	46,07	0,78934	51,9	0,017	-7,07

a) The average molar concentration of dipoles including water and ethanol. N_{av} = density/M_{AV} = density/(x_1M_1+x_2M_2), with x_1 and x_2 mol fraction of solvent 1 and solvent 2, respectively. x_1+x_2 =1. M_1 and M_2 are the molar masses of solvent 1 and 2, respectively.

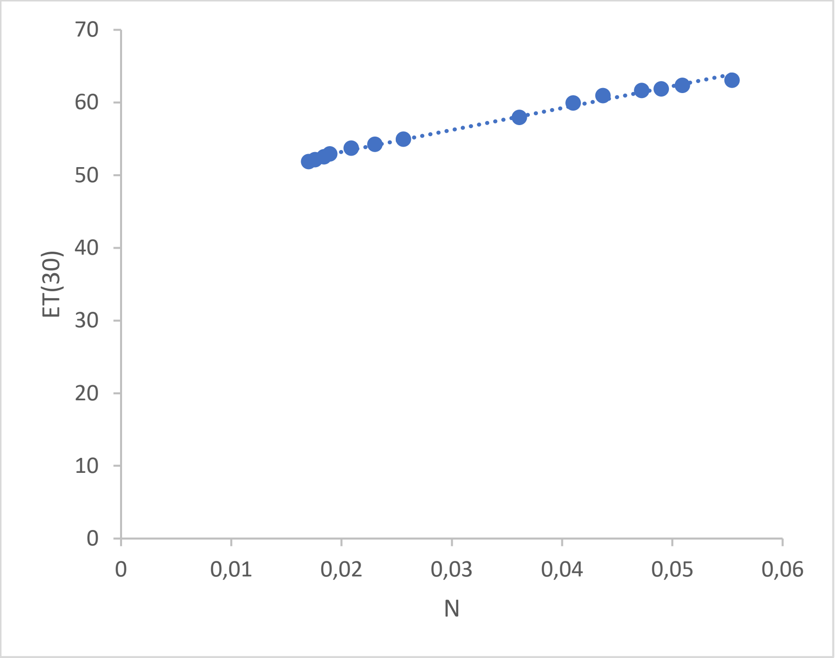

Fig. S 19 Correlation of $E_T(30)$ as function of N(average) for ethanol/water mixture, $E_T(30)$ Data are only from Dimroth, K., Reichardt, C.: Fresenius Z. Anal. Chem. 1966, 215, 344. They were calculated from the given λ_{max} values according to eq. 1 main text. $E_T(30) = 301.55\ N_{av} + 47.173$;
n = 14, r = 0.997.

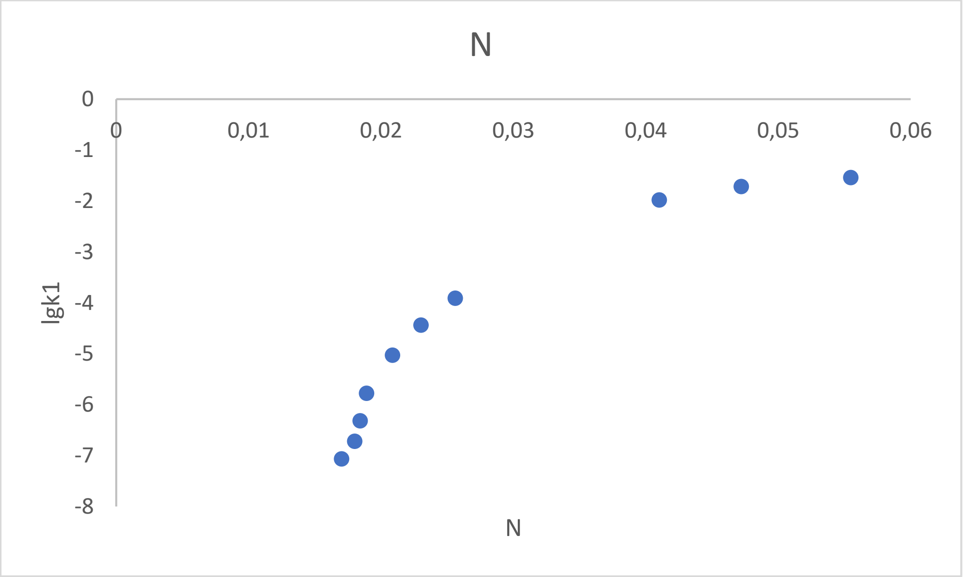

Fig. S20a Plotting lgk_1 as function of N (average) for the ethanol/water solvent system, including the density impact, according to the footnote of Tab. S7.

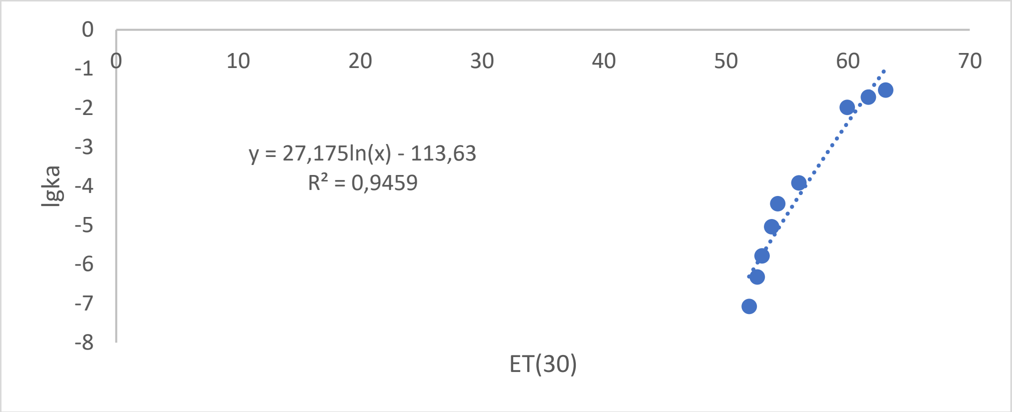

Fig. S20b Correlation of lgk_1 as function of $E_T(30)$ for the ethanol/water solvent system

2.9 Experimental

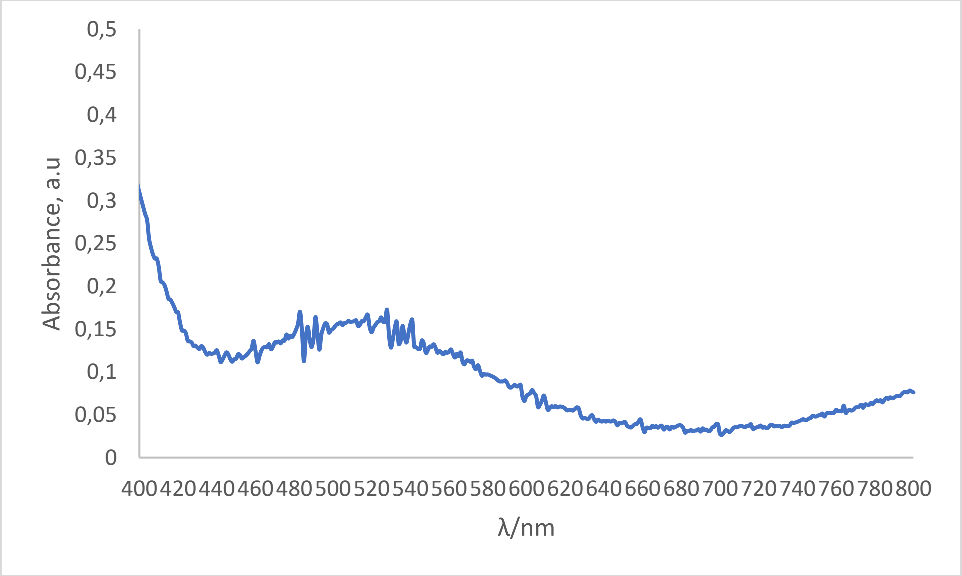

Fig. S21 UV/Vis spectrum of **B30** dissolved in 1,2,4-butanetriol, concentration of B30 is very low.

3. References of the ESI

1) C. J. F. Böttcher, Theory of Electric Polarization (2nd ed.), **1973**, Elsevier. doi:10.1016/c2009-0-15579-4. ISBN 978-0-444-41019-1.

2) a) P. Suppan, *J. Photochem. Photobiol. A*, **1990**, *50*, 293–330; b) P. Suppan and N. Ghoneim, in *Solvatochromism*, Royal Chemical Society, Cambridge, **1997**; c) P. Suppan and C. Tsiamis, *J. Chem. Soc., Faraday Trans. 2*, **1981**, *77*, 1553–1562; d) N. Ghoneim and P. Suppan, *Spectrochim. Acta, Part A*, **1995**, *51*, 1043–1050.
3) S. Spange, N. Weiß, C. Schmidt and K. Schreiter, *Chemistry-Methods*, **2021**, *1*, 42–60.
4) a) N. Weiß, C. H. Schmidt, G. Thielemann, E. Heid, C. Schröder and S. Spange, *Phys. Chem. Chem. Phys*, **2021**, *23*, 1616–1626; b) K. Bica, M. Deetlefs, C. Schröder and K. R. Seddon, *Phys. Chem. Chem. Phys.*, **2013**, *15*, 2703–2711; c) N. Weiß, G. Thielemann, K. Nagel, C. H. Schmidt, A. Seifert, L. Kaßner, V. Strehmel, B. Corzilius, C. Schröder and S. Spange, *Phys. Chem. Chem. Phys.*, **2021**, 23, 26750–26760.
5) N. Kobrak, *Green Chem.*, **2008**, *10*, 80–86.
6) a) H. Langhals, P. Braun, C. Dietl, P. Mayer, *Chem. Eur. J.*, **2013**, *19*, 13511–13521; b) H. Langhals, *Chem. Heterocycl. Compd.*, **2017**, *53*, 2–10.
7) B. Linder, *J. Chem. Phys.*, **1960**, *33*, 668–675.
8) a) L. Onsager, *J. Am. Chem. Soc.*, **1936**, *58*, 1486–1493; b) A. F. Buckingham, *Trans. Faraday Soc.* **1953**, *49*, 881–886; c) W. Schröer, *Ber. Bunsenges. Phys. Chem.*, **1982**, *86*, 916–922; d) G. Bossis, *Molecular Physics*, **1982**, *47*, 1317–1332; e) M. Homocianu, A. Airinei and D. O. Dorohoi, *J. Adv. Res. Phys.*, **2011**, *2*, 011105.
9) M. Born, E. Wolf and A. B. Bhatia, Principles of Optics: Electromagnetic Theory of Propagation, Interference and Diffraction of Light, Cambridge University Press, *1999*.
10) a) R. W. Taft, and M. J. Kamlet, *J. Am. Chem. Soc.*, **1976**, *98*, 2886–2894; b) R. W. Taft, M. J. Kamlet, *J. Chem. Soc. Perk. Trans II*, **1979**, 1979–1729; c) M. J. Kamlet, J.-L. M. Abboud and R. W. Taft, *Prog. Phys. Org. Chem.*, **1981**, *13*, 485–630; d) M. J. Kamlet, J.-L. M. Abboud, M. H. Abraham and R. W. Taft, *J. Org. Chem.*, **1983**, *48*, 2877–2887; e) R. W. Taft, J.-L. M. Abboud, M. J. Kamlet and M. H. Abraham, *J. Sol. Chem.*, **1985**, *14*, 153–186.
11) a) J. Catalán, V. López, P. Pérez, R. Martín-Villamil and J. Rodríguez, *Liebigs Ann. Chem.*, **1995**, 241–252; b) J. Catalán, C. Díaz, V. López, P. Pérez, J.-L. G. De Paz and J. G. Rodriguez, *Liebigs Ann. Chem.*, **1996**, 1785–1794; c) J. Catalán and C. Díaz, *Eur. J. Org. Chem.*, **1999**, 885–891; d) J. Catalán and H. Hopf, *Eur. J. Org. Chem.*, **2004**, 4694–4702; e) J. Catalán, *J. Phys. Chem. B*, **2009**, *113*, 5951–5960; f) J. Catalán and C. Diaz, *Liebigs. Ann.*, **1997**, 1941–1949.
12) a) H. Langhals, *Nouv. Journ. Chim.*, **1982**, *6*, 265–267; b) H. Langhals, *Angew. Chem. Int. Ed.*, **1982**, *21*, 724–733; c) H. Langhals, *Chem. Heterocycl. Compd*. **2017**, 53, 2-10.
13) T. W. Bentley and I. S. Koo, *Org. Biomol. Chem.*, **2004**, *21*, 2376–2380.
14) a) C. Reichardt and T. Welton, in *Solvents and Solvent Effects in Organic Chemistry*, 4th Edition, Wiley-VCH, Weinheim, **2010**; b) ibid, pp. 449; c) ibid, pp. 431.
15) T. G. Mayerhöfer, V. Ivanovski and J. Popp, *Appl. Spectrosc.*, **2021**, *75*, 1526–1531.
16) a) M. You, L. Liu and W. Zhang, *Phys. Chem. Chem. Phys.*, **2017**, *19*, 19420–19426; b) M. You, L. Zhou, X. Huang, Y. Wang and W. Zhang, *Molecules*, **2019**, *24*, 1379.
17) M.P. Wolfshorndl, R. Baskin, I. Dhawan and C. H. Londergan, *J. Phys. Chem. B*, **2012**, *116*, 1172–1179.
18) J. T. Edwards and P. G. Farrell, *Can. J. Chem.*, **1975**, *53*, 2965.
19) a) D. Ben-Amotz and K. G. Willis, *J. Phys. Chem.*, **1993**, *97*, 7736; b) D. Ben-Amotz and D. R. Herschbach, *J. Phys. Chem.*, **1990**, *94*, 1038–1047.
20) Y. Marcus, in *The Properties of Solvents*, 1st Edition, Wiley-VCH, **1998**, pp. 88.
21) G. Graziano, *Biophys. Chem.*, **2003**, *105*, 371–382.
22) M. A. Kessler and O. S. Wolfbeis, *Chem. Phys. Lipids*, **1989**, *50*, 51–56.

23) E. B. Tada, L. P. Novaki and O. A. El Soeoud, *J. Phys. Org. Chem.*, **2000**, *13*, 679–687.
24) a) P. Singh and S. Pandey, *Green Chem.*, **2007**, *9*, 254–361; b) A. Sarkar, S. Trivedi, G. A. Baker and S. Pandey, *J. Phys. Chem. B*, **2008**, *112*, 14927–14936; c) Linear Correlation between E_T(30) and E_T(33) values, compilation made by C. Reichardt, Marburg, **2014** (Handout).
25) K. W. Miller, N. V. Reo, U. Schoot, J. M. Antonius, D. P. Stengle, T. R. Stengle and K. L. Williamson, *Proc. Nat. Acad. Sci.*, **1981**, *78*, 4946–4949.
26) S. M. Rubin, M. M. Spence, A. Pines and D. E. Wemmer, *J. Magn. Reson.*, **2001**, *152*, 79–86.
27) S. Spange, D. Keutel, *Liebigs Ann*. **1993**, 981–985.
28) a) E. Grunewald and S. Winstein, *J. Am. Chem. Soc.*, **1948**, *70*, 864; b) S. Winstein, E. Grunewald and H. W. Jones, *J. Am. Chem. Soc.*, **1951**, *73*, 2700.
29) G. Dvorko, E. Ponomareva, N. Ponomarev, V. Zaliznyi and I. Koshchii, *Rus. J. General. Chem.*, **2007**, *77*, 1535–1558.
30) C. Laurence, S. Mansour, D. Vuluga and J. Legros, *J. Phys. Org. Chem.*, **2020**, *33*, e4067.
31) K.-T. Liu, L.-W. Chang, D.-G. Yu, P.-S. Chen and J.-T. Fan, *J. Phys. Org. Chem.*, **1997**, *10*, 879–884.
32) J. Catalán, *J. Mol. Liq.*, **2021**, *324*, 114699.
33) M. H. Abraham, F. Martins, R. Elvas-Leitão and L. Moreira, *Phys. Chem. Chem. Phys.*, **2021**, *23*, 3311–3320.
34) R. M. C. Gonçalves, A. M. N. Simões and L. M. P. C. Albuquerque, *J. Chem. Soc., Perkin Trans. 2*, **1991**, 825–828.
35) G. Dvorko, V. Zaliznyi and N. E. Ponomareva, *Rus. J. General. Chem.*, **2002**, *72*, 1414–1438.
36) https://www.internetchemie.info/chemie-lexikon/daten/e/ethanol-wasser-dichte.php.
37) K. Dimroth, C. Reichardt, *Fresenius Z. Anal. Chem*. **1966**, 215, 344
38) R. D. Skwierczynski, K. A. Connors, *J. Chem. Soc., Perkin Trans.* 2, **1994**, 467–472
